# SPARC mediates metastatic cooperation between CSC and non-CSC prostate cancer cell subpopulations

**DOI:** 10.1186/1476-4598-13-237

**Published:** 2014-10-21

**Authors:** Francesca Mateo, Óscar Meca-Cortés, Toni Celià-Terrassa, Yolanda Fernández, Ibane Abasolo, Lourdes Sánchez-Cid, Raquel Bermudo, Amaia Sagasta, Leonardo Rodríguez-Carunchio, Mònica Pons, Verónica Cánovas, Mercedes Marín-Aguilera, Lourdes Mengual, Antonio Alcaraz, Simó Schwartz, Begoña Mellado, Kristina Y Aguilera, Rolf Brekken, Pedro L Fernández, Rosanna Paciucci, Timothy M Thomson

**Affiliations:** Department of Cell Biology, Molecular Biology Institute of Barcelona, National Research Council (CSIC), c. Baldiri Reixac 15-21, Barcelona, 08028 Spain; Networking Research Centre for Bioengineering, Biomaterials and Nanomedicine (CIBER-BBN), Instituto de Salud Carlos III, Zaragoza, 50018 Spain; Functional Validation and Preclinical Research, CIBBIM-Nanomedicine, Vall d’Hebron Research Institute, Barcelona, 08035 Spain; Institut d’Investigacions Biomèdiques August Pi i Sunyer (IDIBAPS), Barcelona, 08034 Spain; Department of Pathology, Hospital Clínic, Barcelona, 08034 Spain; Biomedical Research and Translational Oncology Unit, Vall d’Hebron Research Institute, Barcelona, 08035 Spain; Department of Oncology, Hospital Clínic, Barcelona, 08034 Spain; Laboratory and Department of Urology, Hospital Clínic, Barcelona, 08034 Spain; Division of Surgical Oncology, Department of Surgery, Hamon Center for Therapeutic Oncology Research, UT Southwestern Medical Center, Dallas, TX 75229 USA; Universitat de Barcelona, Barcelona, 08034 Spain; Department of Molecular Biology, Princeton University, Princeton, NJ 08544-1014 USA

**Keywords:** SPARC, Tumor heterogeneity, Cell cooperation, Metastasis

## Abstract

**Background:**

Tumor cell subpopulations can either compete with each other for nutrients and physical space within the tumor niche, or co-operate for enhanced survival, or replicative or metastatic capacities. Recently, we have described co-operative interactions between two clonal subpopulations derived from the PC-3 prostate cancer cell line, in which the invasiveness of a cancer stem cell (CSC)-enriched subpopulation (PC-3M, or M) is enhanced by a non-CSC subpopulation (PC-3S, or S), resulting in their accelerated metastatic dissemination.

**Methods:**

M and S secretomes were compared by SILAC (Stable Isotope Labeling by Aminoacids in Cell Culture). Invasive potential *in vitro* of M cells was analyzed by Transwell-Matrigel assays. M cells were co-injected with S cells in the dorsal prostate of immunodeficient mice and monitored by bioluminescence for tumor growth and metastatic dissemination. SPARC levels were determined by immunohistochemistry and real-time RT-PCR in tumors and by ELISA in plasma from patients with metastatic or non-metastatic prostate cancer.

**Results:**

Comparative secretome analysis yielded 213 proteins differentially secreted between M and S cells. Of these, the protein most abundantly secreted in S relative to M cells was SPARC. Immunodepletion of SPARC inhibited the enhanced invasiveness of M induced by S conditioned medium. Knock down of SPARC in S cells abrogated the capacity of its conditioned medium to enhance the *in vitro* invasiveness of M cells and compromised their potential to boost the metastatic behavior of M cells *in vivo*. In most primary human prostate cancer samples, SPARC was expressed in the epithelial tumoral compartment of metastatic cases.

**Conclusions:**

The matricellular protein SPARC, secreted by a prostate cancer clonal tumor cell subpopulation displaying non-CSC properties, is a critical mediator of paracrine effects exerted on a distinct tumor cell subpopulation enriched in CSC. This paracrine interaction results in an enhanced metastatic behavior of the CSC-enriched tumor subpopulation. SPARC is expressed in the neoplastic cells of primary prostate cancer samples from metastatic cases, and could thus constitute a tumor progression biomarker and a therapeutic target in advanced prostate cancer.

**Electronic supplementary material:**

The online version of this article (doi:10.1186/1476-4598-13-237) contains supplementary material, which is available to authorized users.

## Background

The progression from normal tissue to a malignant tumor is driven by the acquisition of genetic and epigenetic changes together with a selection of the cells with an advantage in proliferation and survival [1]. Tumor microenvironments, composed by non-neoplastic cells, can also induce transcriptional reprogramming in neoplastic cells by the secretion of factors like TGF-β and PDGF [2], hormones or hypoxic stress [3] *.* The final outcome is the coexistence in a given tumor of phenotypically different subpopulations or subclones of tumor cells (intratumoral heterogeneity).

Neoplastic cell subpopulations can interact with non-neoplastic elements of the tumor microenvironment and use them for their advantage [4]. In addition, different cell subpopulations within a tumor can interact with each other as in any ecological niche [5], either by competing for common resources [6] or by cooperating for mutual benefit [7, 8]. In this context, interclonal cooperativity can occur, defined as the state in which two or more neoplastic clones display a more malignant phenotype in coexistence than in isolation [9, 10]. Thus, two neoplastic clones - of which one, or both, is not intrinsically invasive and/or metastatic- can interact when they are in proximity to one another in order to become invasive and metastatic.

In a previous study [11], we have characterized clonal subpopulations derived from the PC-3 prostate cancer cell line in which one subpopulation displayed features suggestive of enrichment for CSCs, including high tumorigenic and metastatic potentials, and a second subpopulation was depleted of CSCs and was poorly tumorigenic and metastatic (non-CSC subpopulation). In this model, the CSC-enriched subpopulation shows a strong epithelial phenotype, while, in contrast, the non-CSC subpopulation shows a strong and stable mesenchymal phenotype. We found that the non-CSC subpopulation enhanced the metastatic potential of the CSC-enriched subpopulation [11], thus providing experimental support to the hypothesis of cooperative interactions among CSC and non-CSC tumor cell subpopulations displaying distinct phenotypes [7, 12] with the result of enhanced metastatic dissemination of the overall tumor. Our preliminary evidence also suggested that such cooperation was at least partially mediated by diffusible factors in our cellular models [11]. Here we report that the matricellular protein SPARC is the major diffusible factor produced by the PC-3S non-CSC clonal subpopulation that mediates the enhanced invasiveness and metastatic dissemination of the CSC-rich PC-3M subpopulation of the PC-3 prostate cancer cell line.

## Results

### Neoplastic non-CSC cells enhance the invasiveness of CSC-enriched prostate cancer cells

**Figure 1:**
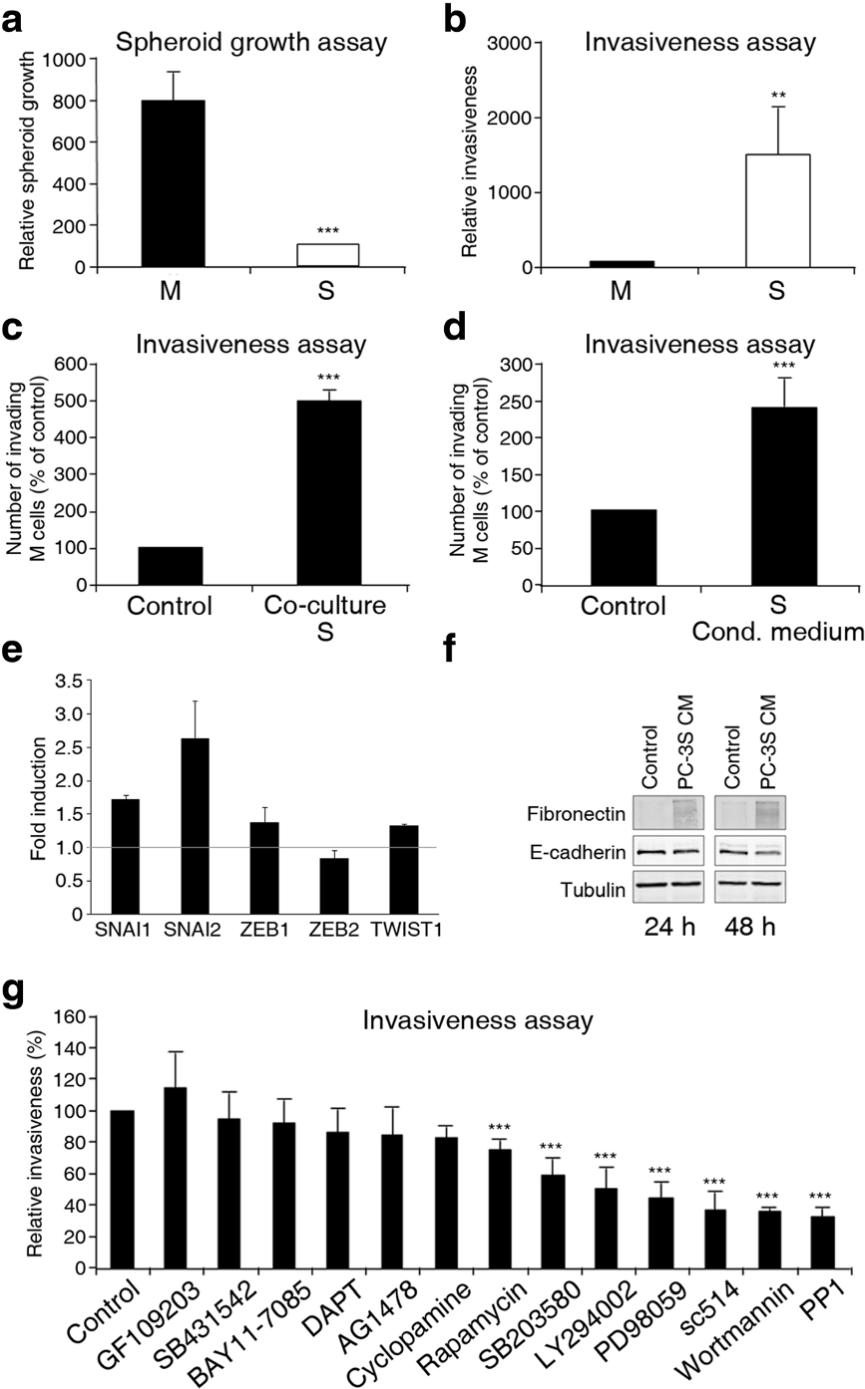
**Conditioned medium from S cells strongly enhance the invasiveness of M cells. (a)** M cells, but not S cells, display a strong potential for anchorage-independent growth. Spheroid assays were performed in triplicates and values shown are mean ± SD. **(b)** S, but not M cells, display a strong intrinsic invasive potential in Transwell-Matrigel assays. **(c)** Co-culture with S cells strongly enhances the invasiveness of M cells. Oregon Green 488-labeled M cells were co-cultured for 24 h with Far Red-DDAO-SE-labeled S, placed on Transwell-Matrigel chambers and invasive cells in the lower chamber scored and assigned cell of origin according to their fluorescence. **(d)** Conditioned medium from S (S-CM) cells strongly enhances the invasiveness of M cells. M cells were treated with control or S-CM and assayed for invasiveness. **(e)** Treatment of M cells for 48 h with S-CM induces the expression of EMT-genes SNAI1, SNAI2, ZEB 1 and TWIST1. Transcript levels for different genes were quantified by real-time RT-PCR. RPL18 (S18) levels were used as internal references and the ΔΔCt method applied to normalize against values determined for M cells treated with CD-CHO control medium **(f)** Treatment of M cells with S-CM induces upregulation of FN1 and downregulation of CDH1. Western blotting experiments with lysates from PC-3M cells treated with S-CM for 24 or 48 h. **(g)** Several signaling pathways are required for the enhanced invasiveness of M cells stimulated by S-CM. M cells were treated with S-CM medium without (control) or with different inhibitors (working concentrations of inhibitors are listed in Additional file 1: Table S1) and assayed for invasiveness. Asterisks: *p* ≤0.001 compared to control. Experiments **(b)** to **(e)** were performed in quadruplicates or triplicates and the data shown are percentages of invasiveness relative to control ± SD. Asterisks denote statistically significant differences (two-tailed Student’s *t*-test).

M and S clonal cell subpopulations were derived from the parental PC-3 prostate cancer cell line [11]. M cells exhibit an epithelial phenotype characterized by cobble-like monolayer growth and the expression of epithelial markers, whereas S cells present a strong mesenchymal phenotype with fibroblast-like morphology and the expression of mesenchymal markers. They also differ in their ability for anchorage-independent growth and invasiveness. Thus, M but not S cells readily form spheroids in *in vitro* 3D cultures, a surrogate indicator of self-renewal potential (Figure 1a). In contrast, S cells exhibit remarkable invasiveness in Transwell-Matrigel assays compared to M cells (Figure 1b).

To determine if the highly invasive S cells can modulate the invasive potential of poorly invasive M cells, we analyzed the invasiveness of M cells alone and after co-culture with S cells. M cells were labeled with Oregon Green 488 carboxy-DFFDA-SE, S cells were labeled with Far Red DDAO-SE, and the two cell lines were seeded in the upper chamber of Transwell-Matrigel units. After 24 h, cells that had invaded to the lower chamber were analyzed by flow cytometry. The results indicated that M cells are significantly enhanced in their invasiveness after co-culture with S cells (Figure 1c and Additional file 1: Figure S1). To distinguish whether the observed effect could be explained by cell-to-cell contact or by diffusible factors, we prepared S-conditioned medium (S-CM) under serum-free growth conditions. As can be seen (Figure 1d), S-CM strongly stimulated the invasiveness of M cells, without major effects on their growth rate (Additional file 1: Figure S2), indicating that diffusible factors secreted by S cells enhance the invasive behavior of M cells.

The incubation of M cells with S conditioned medium caused an induction of the transcript levels of SNAI2 and SNAI1 (Figure 1e) and an upregulation of fibronectin accompanied with a modest downregulation of E-cadherin protein levels (Figure 1f), suggestive of an epithelial-mesenchymal transition, which provides a mechanistic explanation for the enhanced invasiveness observed in M cells. We next explored the relevance of cell signaling cascades in this activity. For this, we treated M cells with S-CM, without (control) or with the addition of inhibitors of selected signaling pathways. Several inhibitors caused 50% or more inhibition of M cell invasiveness induced by S-CM, including the phosphoinositide 3-kinase inhibitors LY294002 and wortmannin, the MAP kinase inhibitor PD98059, the IKK-β inhibitor sc-514, and the Src tyrosine kinase inhibitor PP1 (Figure 1g and Additional file 1: Table S1). Thus, active PI3K, MAPK, NF-κB and tyrosine kinase pathways are required for an optimal invasive response by M cells to S-CM.

### SPARC is the extracellular protein most abundantly secreted by S cells *vs*. M cells

**Figure 2:**
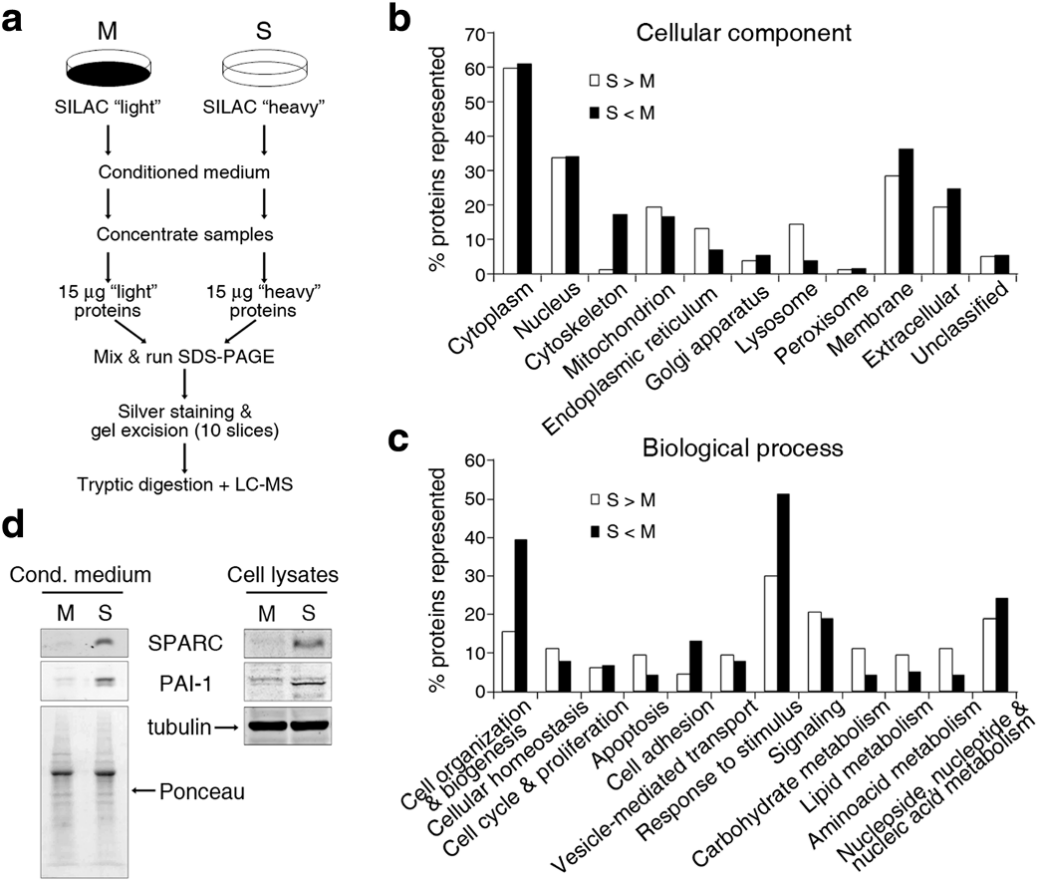
**SPARC is the secreted protein most abundantly produced by S cells relative to M cells. (a)** Schematic depiction of the procedure used to identify the proteins present in M-CM and S-CM using SILAC labeling. **(b)** Gene Ontology and Uniprot subcellular localization predictions for the differentially secreted proteins. **(c)** Gene Ontology biological process predictions for the differentially secreted proteins. S > M*:* proteins with H/L ≥2. S < M*:* proteins with H/L ≤ -2. **(d)** Western blotting analysis of cell extracts and conditioned medium confirms the differential expression of SPARC and PAI-1 between S and M cells. Tubulin signal was used as a protein loading and transfer control for cell lysates, and Ponceau red staining for conditioned medium.

We used SILAC for an unbiased identification of components of S-CM potentially responsible for the enhanced invasiveness of M cells (Figure 2a). S cells were metabolically labeled with “heavy” forms of L-lysine and L-arginine (^13^C_6_-L-lysine and ^13^C_6_-L-arginine), whereas M cells contained the “light” forms of the same amino acids. Conditioned media from both cell lines were mixed at a 1:1 ratio, subjected to SDS-PAGE, silver stained, eluted, digested, peptides identified by mass spectrometry and their relative levels determined as the ratio between “heavy” *vs*. “light” peptides (Additional file 2: Table S2). We considered the proteins identified with a heavy/light (H/L) ≥2 ratio as differentially secreted by S cells (over-represented in S cells), whereas proteins with a H/L ≤ -2 ratio were considered as secreted predominantly by M cells (under-represented in S cells). Putative subcellular localizations of the identified proteins were assigned with the aid of the Gene Ontology and UniProtKB databases (Figure 2b). Several proteins are assigned more than one subcellular compartment, which explains that the sum of all compartments may exceed 100%. As described for other cellular secretomes [13–15], the majority of the proteins identified in the conditioned media from both M and S cells were assigned a cytoplasmic localization, and only about 20% of the proteins were predicted extracellular or secreted status. Cytoskeletal proteins were more abundant in M-CM than in S-CM, whereas lysosomal proteins were more abundant in the latter (Figure 2b). Gene Ontology and UniProtKB databases were also used to functionally classify the identified proteins. The proteins over-represented in S-CM are mainly involved in apoptosis and carbohydrate, lipid and amino acid metabolism, whereas those over-represented in M-CM are mainly involved in cell adhesion, cell organization and biogenesis and response to stimulus (Figure 2c).

We reasoned that the diffusible factor or factors responsible for the increased invasiveness of M cells in response to S-CM would most likely reside within the set of canonical extracellular proteins over-represented in S-CM. Of these, SPARC ranged first, with a H/L ratio of 13.32, followed by PAI-1 (Plasminogen Activator Inhibitor-1), Extracellular Superoxide Dismutase, Calreticulin and Pentraxin-3 (Table 1). These five top-scoring differentially secreted proteins are of particular interest because they are potential markers of cancer progression and metastasis and some of them play roles in cell migration, wound healing and invasion [14, 16–21]. Quantitative real-time PCR (qPCR) analysis indicated that their corresponding mRNAs are indeed over-represented in S cells relative to M cells, with SPARC and PAI-1 expression levels in S cells more than ten-fold higher than in M cells (Additional file 1: Figure S3). The abundance of these two proteins in cell extracts and conditioned medium was also analyzed by Western blotting, confirming that SPARC and PAI-1 are expressed and secreted at significantly higher levels by S cells than M cells (Figure 2d). The expression levels of SPARC in parental PC-3 cells were intermediate between the relatively high levels in S cells and the lower levels in M cells (Additional file 1: Figure S4). SPARC expression levels were extremely low or undetectable in the androgen-independent Du-145 and CWR22Rv1 (henceforth, 22Rv1) prostate cancer cells and in the androgen-dependent LNCaP cells (Additional file 1: Figure S4).

**Table 1:** **List of extracellular proteins most significantly overrepresented in S** ***vs*** **. M conditioned medium**

Accession	Protein name	H/L ratio	Expression in microarrays
SPRC_HUMAN	SPARC	13.32	S > M
PAI1_HUMAN	Plasminogen activator inhibitor 1 = SERPINE1	12.81	S > M
SODE_HUMAN	Extracellular superoxide dismutase [Cu-Zn] OS = SOD3	11.11	S > M
CALR_HUMAN	Calreticulin = CALR	10.92	NOT CONCLUSIVE
PTX3_HUMAN	Pentraxin-related protein PTX3 = PTX3	7.33	S > M
SODC_HUMAN	Superoxide dismutase [Cu-Zn] = SOD1	5.92	S > M
ISG15_HUMAN	Ubiquitin-like protein ISG15 = ISG15	4.10	S > M
PDIA1_HUMAN	Protein disulfide-isomerase = P4HB	3.84	S > M
CS010_HUMAN	UPF0556 protein C19orf10 = C19orf10	3.81	S > M
ERAP1_HUMAN	Endoplasmic reticulum aminopeptidase 1 = ERAP1	3.61	NOT PRESENT IN MA*
CATZ_HUMAN	Cathepsin Z = CTSZ	3.38	NOT CONCLUSIVE
GGH_HUMAN	Gamma-glutamyl hydrolase = GGH	3.32	S > M
TCTP_HUMAN	Translationally-controlled tumor protein = TPT1	2.56	S > M
PEBP1_HUMAN	Phosphatidylethanolamine-bindingprotein 1 = PEBP1	2.46	M > S
AIBP_HUMAN	Apolipoprotein A-I-binding protein = APOA1BP	2.23	NOT CONCLUSIVE

*MA, Microarray.

### SPARC mediates the enhanced invasiveness of M cells stimulated by S cells

**Figure 3:**
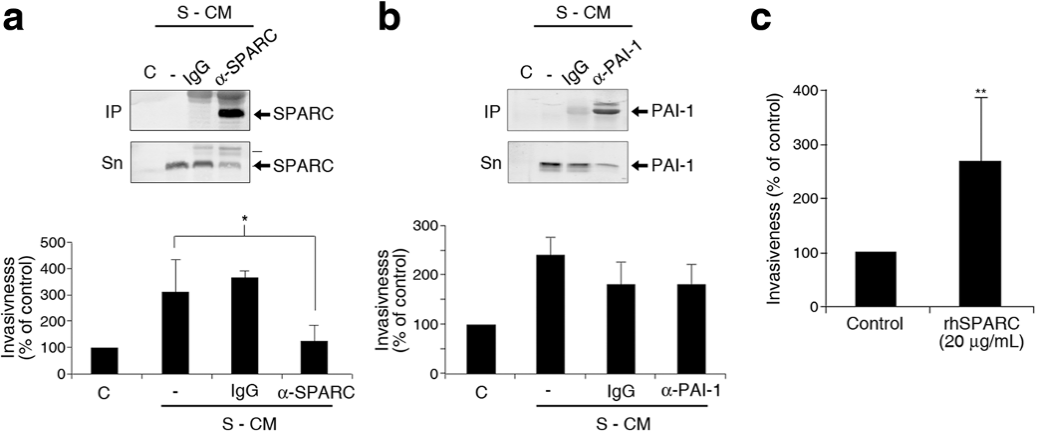
**Immunodepletion of SPARC abrogates the M pro-invasive activity of S-conditioned medium. (a)** Immunodepletion of SPARC abrogates the ability of S conditioned medium to enhance the invasiveness of M cells. Top panel: Western blots show the amount of SPARC protein present in immunoprecipitates (IP) and supernatants (Sn) of S conditioned medium subjected to immunoprecipitation of SPARC using 2 μg/mL of anti-SPARC plus protein G (2 h incubation at 4°C). IgG plus protein G was used as a specificity control. C corresponds to control medium and (-) corresponds to S-CM treated only with protein G beads but no specific antibodies. Bottom panel: Relative invasiveness in Transwell-Matrigel assays of M cells treated with control medium, S-conditioned medium, and medium immunodepleted for SPARC or its IgG control. **(b)** Same as in **a**, substituting a specific anti-SPARC antibody for anti-PAI-1. **(c)** Exogenous SPARC stimulates the invasiveness of M cells. Transwell-Matrigel assay comparing the relative invasiveness of M cells under standard conditions or treated with 20 μg/mL of recombinant human SPARC protein. All experiments were performed in quadruplicates or triplicates and the data shown are percentages of invasiveness relative to control ± SD. Asterisks denote statistically significant differences (two-tailed Student’s *t*-test).

To determine the importance of SPARC in the pro-invasive activity of S-CM on M cells, we performed invasion assays comparing M cells alone, M cells treated with S-CM and M cells treated with S-CM that had been depleted of SPARC using a specific antibody. Immunodepletion of SPARC from S-CM abrogated its ability to enhance the invasive behavior of M cells (Figure 3a). In parallel experiments, immunodepletion of PAI-1 from S-CM did not significantly inhibit its ability to enhance the invasion of M cells (Figure 3b). Furthermore, the addition of recombinant SPARC to the culture medium enhanced the invasive behavior of M cells (Figure 3c). These observations allow us to conclude that SPARC, but not PAI-1, is a candidate secreted factor that may explain the pro-invasive effect of S-CM on M cells.

On the other hand, time-course experiments showed that incubation with SPARC induced the phosphorylation of ERK1/2 in M cells (Additional file 1: Figure S5), suggesting a role for the activation of the MAPK pathway in the observed phenotypic effects caused by SPARC, also supported by the abrogation of S-CM-stimulated invasiveness of M cells by MAPK inhibitors described above (Figure 1g). Although the activation of AKT by SPARC is less conspicuous because of the basal levels of activation of the PI3K-AKT pathway due to the loss of PTEN in PC-3 cells [22], the participation of this pathway in the effects induced by SPARC in M cells is also supported by the above experiments with pathway component inhibitors (Figure 1g). Of interest, ILK, expressed at low levels in M cells, was not activated in these experiments (Additional file 1: Figure S5). This suggests that the integrin-ILK pathway, shown by others to couple SPARC-prompted signaling in other models [23–25], is not involved in the invasive response of M cells to SPARC.

**Figure 4:**
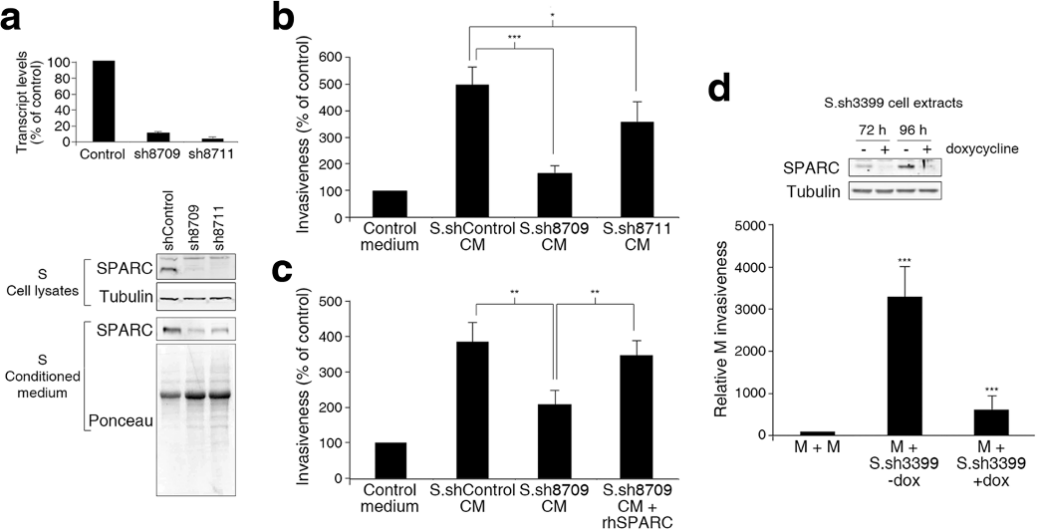
**SPARC is a key factor mediating the pro-invasive activity of S cells on M cells. (a)** Validation of SPARC knockdown in S cells. Top: qPCR transcript quantification of SPARC in S cells transduced with SPARC-targeting (sh8709 and sh8711) or control shRNAs. RPL18 (S18) levels were used as internal references. Values are plotted as percent transcript levels in SPARC-knockdown *vs*. control cells. Bottom: Western blotting verification of SPARC knockdown by sh8709 and sh8711. Tubulin signal was used as loading and transfer reference for cell lysates, and Ponceau red staining for conditioned medium. **(b)** Knock down of SPARC in S cells abrogates their conditioned medium pro-invasive activity on M cells. Transwell-Matrigel assays compared the relative invasiveness of M cells treated with conditioned medium from S.shControl, S.sh8709 or S.sh8711 cells. **(c)** Recombinant SPARC restores the pro-invasive activity of conditioned medium from S.sh8709 cells. Transwell-Matrigel assays compared the relative invasiveness of M cells treated with conditioned medium from S.shControl, S.sh8709 or S.sh8709 cells supplemented with 20 μg/mL recombinant human (rh) SPARC. M invasiveness under rhSPARC-supplemented S.sh8709-conditioned medium was not significantly different from that induced by control medium. **(d)** SPARC is required for the induction of M invasiveness by co-cultured S cells. Top: S cells integrating doxycycline-inducible SPARC-specific shRNA (S.sh3399) were treated, or not, with 1 μg/mL doxycycline for 72 or 96 h and analyzed for SPARC levels by Western blotting. Tubulin was used as a loading and transfer reference. Bottom: Transwell-Matrigel assay comparing the relative invasiveness of M.GFP cells after 24 h of co-culture with control M cells or S.sh3399 cells treated, or not, with 1 μg/mL doxycycline. Invasiveness experiments in **(b)**, **(c)** and **(d)** were performed in triplicates or quadruplicates and shown are percentages of invasiveness relative to control ± SD. Asterisks denote statistically significant differences (two-tailed Student’s *t*-test).

We next proceeded to knock down SPARC in S cells by means of specific shRNAs, yielding S.sh8709 cells. Knock down of SPARC was verified by qPCR and Western blotting of cell extracts and conditioned medium (Figure 4a). Conditioned medium from S.sh8709 cells showed a reduced ability to enhance the invasiveness of M cells as compared to control S-CM (Figure 4b). Moreover, addition of purified recombinant human SPARC to S.sh8709 conditioned medium rescued its ability to enhance the invasiveness of M cells, restoring it to a pro-invasive activity comparable to that of control S-CM (Figure 4c). Of note, knockdown of SPARC in S cells caused a decrease in their invasiveness (Additional file 1: Figure S6). We also tested the effect of SPARC on the invasive potential of other prostate cancer cells, finding that recombinant human SPARC caused an enhanced invasiveness of the androgen-independent Du-145 and 22Rv1 cells, but not the androgen-dependent LNCaP cells (Additional file 1: Figure S7). Taken together, these results indicate that SPARC is the major factor responsible for the enhanced invasiveness of M cells stimulated by S-CM, and that it can stimulate the invasiveness of other androgen-independent prostate cancer cells.

Of additional interest, knock down of SPARC in S cells was accompanied with a decrease in PAI-1 expression at the mRNA and protein levels (Additional file 1: Figure S8). It has been reported that the expression of PAI-1 is modulated by changes in the expression levels of SPARC [26]. Therefore, although our immunodepletion experiments suggest that PAI-1 may not play a major role in the enhanced invasiveness of M cells by S-CM, we cannot completely rule out the involvement of PAI-1 in the cooperative interaction between our tumor cell subpopulations, albeit secondary to modulation of SPARC levels.

In order to determine the relative importance of SPARC in the M and S cell interactions through diffusible factors or direct cell-to-cell contact, we performed a co-culture experiment, in which M cells were mixed and cultured together, in a 1:1 proportion, with S cells bearing a construct for the doxycycline-inducible expression of a SPARC-specific shRNA (S.sh3399) (Figure 4d and Additional file 1: Figure S9). Under control conditions, S cells significantly enhanced the invasiveness of M cells (Figure 4d). Importantly, doxycycline-induced SPARC knock down abrogated the capacity of S cells to enhance the invasive behavior of M cells (Figure 4d). These results indicate that the expression of SPARC by S cells explains not only their ability to enhance the invasiveness of M cells through secreted factors, but also the overall pro-invasive properties of S cells on M cells, encompassing effects mediated by diffusible factors and by putative direct cell-to-cell contact.

### SPARC produced by non-CSC S cells boosts the *in vivo*growth and metastatic dissemination of CSC-enriched M cells

**Figure 5:**
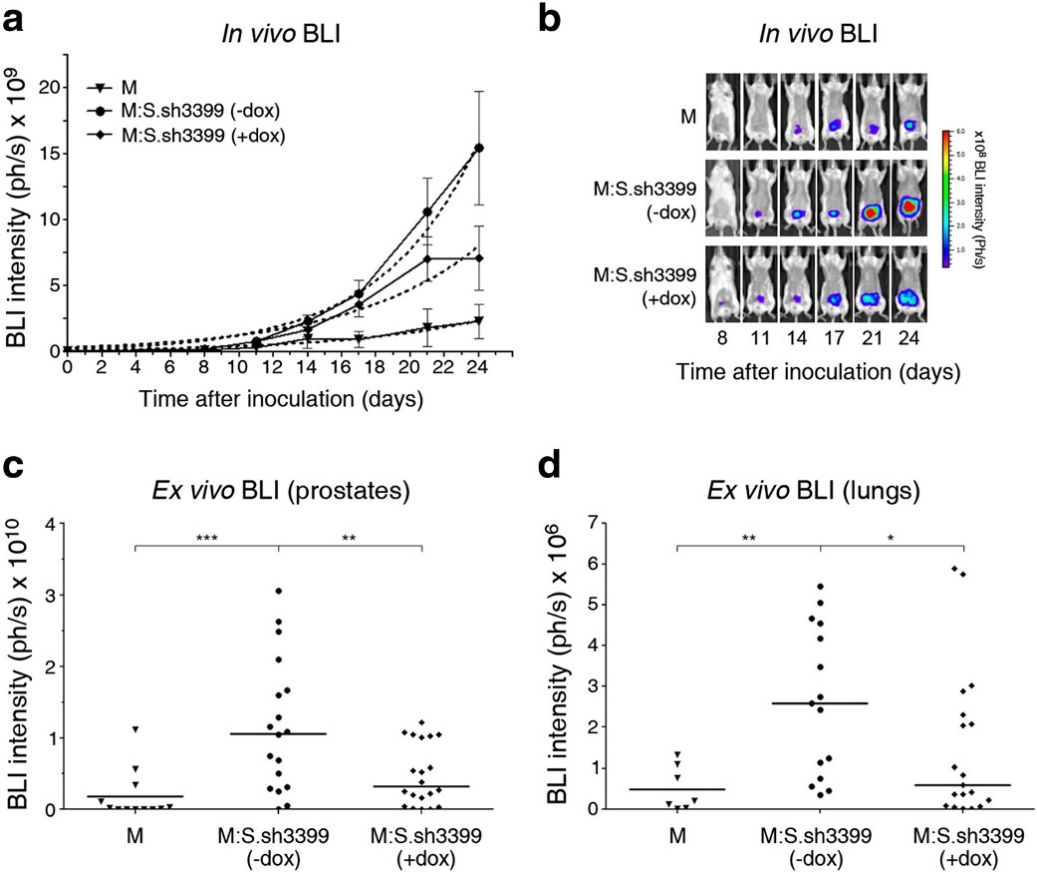
**SPARC boosts** ***in vivo*** **growth and metastatic dissemination of M cells. (a)** SCID-Beige mice (n =10 / experimental group) were inoculated orthotopically (dorsal prostate) with 1x10^5^ firefly luciferase-expressing M.Fluc and S.Fluc.sh3399 cells, as indicated, and growth monitored by external BLI. Mice implanted with S.Fluc.sh3399 cells integrating doxycycline-inducible SPARC-specific shRNA were administered, or not, doxycycline for the entire duration of monitoring (+dox or -dox, respectively). **(b)** Representative images corresponding to mice in the same experimental groups as in **(a). (c, d)** Upon termination of the *in vivo* bioluminescent monitoring (day 24), prostates (**c**) and lungs **(d)** from inoculated animals were removed, incubated with D-luciferin and photon counts determined. Asterisks denote statistically significant differences (two-tailed Mann–Whitney test). Dotted lines represent non-linear fitting to the exponential growth equation Y = Y_0_^(kX)^.

Our previous studies [11] had indicated that the metastatic potential of CSC-enriched M cells is strongly boosted by co-implantation in immunodeficient mice together with non-CSC S cells. To determine the importance of SPARC expressed by S cells in their pro-metastatic cooperation with M cells, we proceeded to the implantation of M cells together with S.sh3399 cells in the dorsal prostates of male SCID-beige mice. Tumor growth at the site of implantation and spread to distant organs was monitored by bioluminescent imaging (BLI), with or without doxycycline in the drinking water of mice. Without doxycycline administration, co-implantation of M cells and S.sh3399 cells strongly accelerated tumor growth at the orthotopic implantation site as compared to the growth rate of M cells alone (Figure 5a). Remarkably, administration of doxycycline, which suppresses SPARC expression in S.sh3399 cells (Figure 4d), significantly inhibited the tumor-stimulating effect of S.sh3399 cells on M cells (Figure 5b and c). In control experiments, S.sh3399 cells alone did not grow detectable tumors or metastases 24 days after implantation in the prostate (Additional file 1: Figure S10), and doxycycline alone did not inhibit the growth of M cells (Additional file 1: Figure S11). This suggests that SPARC produced by S cells is a major inducer of the accelerated growth of M cells upon co-implantation with S cells in the prostate.

We next quantified the luciferase activity in the lungs from mice with prostatic implantation of M cells, together or not with S.sh3399 cells, with or without doxycycline administration. Lungs were removed from euthanized mice at day 24 of monitoring for *ex vivo* bioluminescence quantification. It can be seen that co-implantation of M cells with S.sh3399 cells, without doxycycline administration, stimulated the localization in the lungs of light-emitting tumor cells (Figure 5d). Histochemical examination confirmed that these lungs effectively harbored tumor cell colonies (Additional file 1: Figure S12). Administration of doxycycline strongly inhibited this effect (Figure 5d), indicating that SPARC produced by S cells is responsible for the enhanced metastatic spread of M cells stimulated by S cells.

### SPARC is expressed in the epithelial tumoral components of metastasis-associated primary prostate tumors

**Figure 6:**
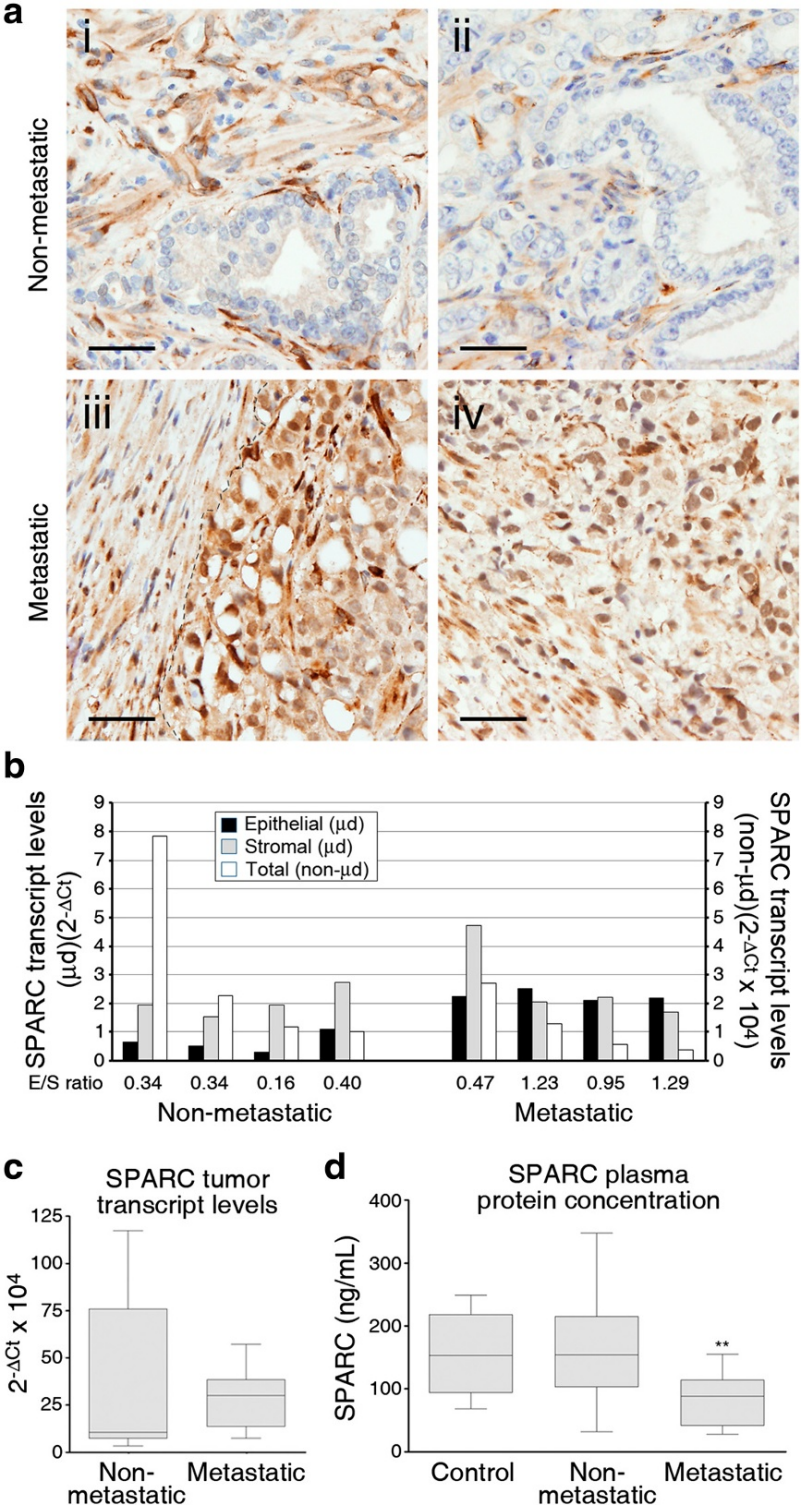
**Increased expression of SPARC in the epithelial component of primary prostate cancer associated with lymph node metastasis. (a)** Immunohistochemical staining for SPARC of primary prostate cancer samples without (non-metastatic; samples i and ii) or with (metastatic; samples iii and iv) associated lymph nodes. A discontinuous line in sample iii delimits the stromal and epithelial tumoral components. Images are representative of stainings performed on 14 non-metastatic and 16 metastatic primary prostate cancer samples (case ID’s 28, 31, 38 and 59 from Additional file 1: Table S4). Size bar, 100 μm. **(b)** Real-time RT-PCR determinations of SPARC transcript levels in laser-microdissected epithelial or stromal components of non-metastatic or metastatic primary prostate cancer samples. Values represent transcript levels inferred from Ct values for SPARC normalized to Ct values for reference β_2_-microglobulin transcripts. Overall, non-microdissected transcript levels for the corresponding samples, determined in **(c)**, are represented next to the relative levels for the microdissected components. Data are expressed as 2^-ΔCt^ (microdissected samples, TaqMan assays) or 2^-ΔCp^ (non-microdissected samples, UPL assays) using β_2_-microglobulin or 18S ribosomal RNA levels, respectively, as internal references. Note that value scales are different for microdissected (μd, left y-axis) or non-microdissected (non-μd, right y-axis) samples. **(c)** Real-time RT-PCR determinations of SPARC transcript levels in non-metastatic (n =15) or metastatic (n =17) primary prostate cancer samples. Data are expressed as 2^-ΔCp^, using 18S ribosomal RNA levels as an internal reference. No statistically significant differences are observed in median levels for non-metastatic *vs*. metastatic samples. **(d)** Plasma SPARC levels determined by ELISA in control individuals (n =4) and in patients with non-metastatic (n =10) or metastatic (n =14) prostate cancer. Asterisks denote statistically significant differences in median plasma SPARC levels between patients with metastatic prostate cancer and patients with non-metastatic disease or controls (two-tailed Mann–Whitney test).

Our above results suggest that expression and secretion of high levels of SPARC by a non-CSC neoplastic cell subpopulation promote the metastatic spread of tumor cells with CSC traits. Conflicting reports have described the expression levels of SPARC to be either increased or decreased in association with advanced prostate cancer [18, 27]. We thus performed an immunohistochemical analysis of SPARC expression in primary prostate carcinoma samples associated, or not, with lymph node involvement (metastatic or non-metastatic primary tumors, respectively). The specificity for SPARC of the antibody used in these analyses was thoroughly verified in the preceding RNAi-mediated knockdown, immunoprecipitation and Western blotting experiments. This analysis showed that SPARC was readily detectable in the stromal components of all tumors, except one metastatic case, without evident associations of percentages or intensities of positive cells with metastatic status (Figure 6a, Additional file 1: Table S4). Remarkably, 14 out of 16 primary metastatic tumors, and 1 out of 14 non-metastatic tumors analyzed, also displayed unequivocal staining for SPARC in their epithelial tumoral components. The proportion of epithelial cells that stained for SPARC varied from 10% to 100% in different samples, ranging in intensity from weak to strong, mostly with a cytoplasmic pattern but also with a clear nuclear staining in 10 out of the 16 samples from metastatic cases (Figure 6a, Additional file 1: Table S4). In spite of its predominant nuclear localization in several metastatic cases, SPARC staining also consistently yielded discrete dots localized in the cytoplasm, with a perinuclear or juxtamembrane patterns (Figure 6a). The staining for SPARC in the epithelial tumoral component correlated with high Gleason scores of the samples (Additional file 1: Table S4), which agrees with the known association of metastatic prostate cancer with high Gleason indexes of primary tumors [28].

The observed staining for SPARC in the epithelial tumoral component of prostate cancer samples could be attributed either to endogenous SPARC produced by tumor cells, or to stromally produced SPARC uptaken by neoplastic cells. Others have shown that exogenous SPARC can be internalized by cells [29, 30] and, indeed, M cells incubated with S-CM as a source of SPARC show enhanced cytoplasmic and nuclear staining for SPARC, suggesting active uptake (Additional file 1: Figure S13). Thus, in order to further establish the origin of the observed staining for SPARC in prostate tumor samples, real-time PCR was performed on RNA samples isolated from laser-microdissected epithelial or stromal components of non-metastatic or metastatic primary tumors. The epithelial components of tumors from the four non-metastatic cases expressed lower levels of SPARC mRNA than the corresponding stromal components (epithelial/stromal ratios of 0.16, 0.34, 0.34 and 0.40) (Figure 6b). In contrast, the epithelial components of tumors from all four metastatic cases expressed higher SPARC mRNA levels than the epithelial components of non-metastatic cases, surpassing in several cases the relative expression levels of the corresponding stromal components (epithelial/stromal ratios of 0.47, 0.95, 1.29 and 1.29) (Figure 6b). This increased expression of SPARC in the epithelial tumor components of metastatic cases did not appear to associate with higher SPARC expression levels in the overall tumor sample, as tumors with low overall levels exhibited high epithelial/stromal SPARC expression ratios and tumors from non-metastatic cases with high overall SPARC expression levels showed low epithelial/stromal SPARC expression ratios (Figure 6b). These observations support the endogenous neoplastic cell of origin of the SPARC staining observed in the epithelial tumoral components of primary prostate tumors associated with metastasis, although they do not formally rule out a stromal contribution of the SPARC protein detected in the neoplastic cells.

In contrast to the strong concordance between epithelial tumor expression of SPARC and metastatic status, overall SPARC mRNA levels from non-microdissected samples did not show a significant correlation with metastatic status (Figure 6c), with the observation of a high variability among samples, in particular in non-metastatic cases. To determine if elevated epithelial SPARC levels in metastasis-associated primary tumors is reflected in levels of circulating SPARC, we quantified by ELISA the levels of SPARC in the plasma of prostate cancer patients with our without associated lymph node or distant metastases that were not under systemic therapy. We found that plasma SPARC levels in metastatic prostate cancer patients tended to be lower than those in non-metastatic cases or control individuals (Figure 6d), and thus we conclude that plasma levels of SPARC in prostate cancer patients do not reflect tumoral SPARC expression levels.

To summarize these observations, expression of SPARC is limited to the stroma of non-metastatic primary tumors, whereas primary tumors from metastatic cases also express SPARC in the tumoral epithelium. Overall tumor SPARC mRNA levels or plasma SPARC protein levels show no significant correlation to metastatic status in prostate cancer.

## Discussion

Neoplastic cellular subpopulations displaying distinct phenotypes can interact among them in different ways [31], including competition [6, 32] or cooperation [33]. In a prostate cancer cell model previously characterized by us [11], two subpopulations interact in a cooperative manner, such that one subpopulation enhances the metastatic dissemination potential of the other. In this model, a clonal tumor cell subpopulation displays non-CSC characteristics, including poor anchorage-independent growth *in vitro* and limited tumorigenic or metastatic potential *in vivo*, whereas a second subpopulation is enriched in CSCs as inferred from vigorous anchorage-independent growth and tumor formation and lung colonization potential *in vivo*. We had previously found that the non-CSC subpopulation enhances the ability of the CSC subpopulation for metastatic dissemination, and produced preliminary evidence that this activity involved, at least in part, diffusible factors secreted by S cells [11]. In the present study, we have confirmed and extended our original observations, including the induction of epithelial-mesenchymal transition in the CSC-enriched epithelial PC-3M subpopulation upon culture with conditioned medium from the non-CSC PC-3S subpopulation, and the dependence of this effect on specific signaling pathways.

We have applied a comparative proteomics approach in an effort to identify proteins differentially secreted by S (non-CSC) *vs*. M (CSC) cells as candidates to explain the observed paracrine effects. Immunodepletion, specific transcript knockdown and complementation experiments have led us to conclude that, of the secreted proteins with the strongest differential secretion between S and M cells, the matricellular protein SPARC, abundantly secreted by S cells, explains most of the pro-invasive effects of S conditioned medium on M cells. Knock down of SPARC abrogated not only the pro-invasive activity of the conditioned medium from the non-CSC S subpopulation on the CSC-enriched M cells, but also the overall pro-invasive and pro-metastatic activities observed upon co-culture *in vitro* or co-inoculation *in vivo*. Of note, conditioned medium from non-CSC S cells required specific signaling pathways for the enhancement of the invasive behavior of M cells, including PI3K, MAPK, NF-κB and non-receptor tyrosine kinase pathways, which are also involved in cellular responses elicited by SPARC [34]. The observed induction by SPARC in M cells of the MAPK and PI3K-AKT signaling pathways further supports the role played by these pathways in our model. This underlines the relevance of paracrine interactions between tumor cell subpopulations displaying non-CSC and CSC properties to modulate the phenotypic outcomes of the tumor. Other laboratories performing unbiased secretome analyses have shown that SPARC also mediates cooperation between tumor cell subpopulations with different invasive potentials in ovarian and bladder cancer models [17, 35].

Although our knockdown and complementation experiments strongly support that SPARC is indeed the key factor mediating invasive and metastatic cooperation in our interacting CSC *vs*. non-CSC neoplastic subpopulation model, other molecules produced by neoplastic or non-neoplastic cells, such as PAI-1, could also participate in this process. In this regard, we observed that knockdown of SPARC in S cells was accompanied with a decrease in PAI-1 expression. It is known that the expression of PAI-1 is modulated by changes in the expression levels of SPARC [26]. Our subsequent immunodepletion experiments suggest that PAI-1 may not play a major role in the enhanced invasiveness of M cells by S-CM. However, additional experiments may be required to completely rule out the involvement of PAI-1 in the cooperative interaction between our tumor cell subpopulations, albeit secondary to modulation of SPARC levels.

SPARC, also known as osteonectin or BM-40, is a matricellular glycoprotein expressed in a variety of tissues during development and tissue repair and remodeling that regulates extracellular matrix deposition and cell-matrix interactions [36]. SPARC has been shown to regulate cell proliferation, cell rounding, cell adhesion, angiogenesis, extracellular matrix remodeling and tumorigenesis, and to be involved in epithelial-mesenchymal transition. In cancer, SPARC has been reported to exert apparently contrasting activities [37–39], either stimulating or inhibiting cell migration and invasion, promoting or reducing tumor growth and metastatic dissemination, sustaining cell survival or causing apoptosis, senescence and sensitization to genotoxic drugs. This suggests that the outcomes of activities mediated by SPARC may be context-dependent. Relevant variables include the source of SPARC (tumoral, stromal, or hematopoietic cells) with differences in glycosylation or peptide fragment patterns that result in functional differences, activities mediated by intracellular *vs*. extracellular SPARC, or the status of pathways regulated by p53 or PTEN [34]. The contrasting activities displayed by SPARC in different normal and neoplastic cell types are reflected in its varying status in different tumors [40]. Thus, SPARC expression is elevated in many tumor types, including prostate cancer [27], and it has been found to enhance the migration and invasiveness of prostate cancer cells [41, 42]. In contrast, other studies have reported downregulation of SPARC in several tumor types, often in association with promoter hypermethylation, and its expression levels to be negatively correlated with tumor stage, therapeutic response or patient outcome. The importance of the interactions between neoplastic cells and tumoral environment is highlighted by the associations of stromal expression of SPARC with tumor progression and patient outcome [43].

We have found that both primary prostate cancer samples associated with metastasis and those without metastatic association express SPARC in their stromal components at variable levels that do not correlate with metastatic status. However, our immunohistochemical analysis has revealed that only those primary tumors associated with metastasis express significant levels of SPARC in their epithelial tumoral components. The origin of this SPARC in the neoplastic component is supported by laser microdissection and RNA quantification experiments that allow us to distinguish the epithelial-tumoral *vs*. stromal origin of SPARC and show that SPARC immunostaining in the epithelial tumoral component correlates with the expression of relatively high mRNA levels in the tumoral component. The observed staining pattern was generally cytoplasmic, perinuclear or juxtamembrane dots, but also a diffuse nuclear staining in a significant proportion of cells in over half of the metastatic primary tumor samples analyzed. A nuclear localization of SPARC has long been noted [44, 45] and, although the potential functions of this nuclear form of SPARC are unknown, it has been linked to cellular proliferation [44]. The observed enhanced epithelial/stromal ratios of SPARC expression levels in metastasis-associated primary tumors did not result in increased overall SPARC transcript levels in the same samples or in plasma SPARC protein levels. These observations, along with those from our *in vitro* and mouse xenograft cell models, underscore the importance of tumor-produced SPARC over that produced by stromal cells in conferring a metastatic phenotype to prostate cancer tumor cells.

There is consistent experimental evidence that the extracellular matrix remodeling, antiangiogenic and antiproliferative effects mediated by non-tumoral SPARC, produced by stromal fibroblasts or endothelial or hematopoietic cells, constitutes a barrier to tumor progression, invasion and metastasis in mouse models of lung, ovarian, bladder, prostate or pancreas cancers [43, 46–50]. Such a conclusion is in line with a potentially more general anti-metastatic function of tumoral stroma, as highlighted by recent observations [51, 52] that are prompting a revision of prior notions on the role of the tumor microenvironment in modulating the metastatic behavior of cancer cells [53]. In parallel, there is also substantial evidence that SPARC endogenously produced by cancer cells favors their invasive, survival and tumorigenic properties [24, 54–62]. The relevance in human tumor progression of a cell-autonomous pro-metastatic function of SPARC is supported by its expression by the cancerous components of metastatic primary tumor samples of glioblastoma, melanoma and prostate cancer, but not in samples from non-metastatic primary tumors [18, 40, 55, 61].

Few experimental models have simultaneously addressed the role of both cancerous and non-cancerous SPARC in tumor progression. A TRAMP mouse model crossed to SPARC^-/-^ mice [50] concluded that both cancerous and non-cancerous SPARC exert tumor suppressor functions. Our results, however, suggest a different perspective in which (1) a relevant factor may be the interaction between SPARC-producing cancerous subpopulations, which are non-metastatic in our model (S cells), with SPARC-responding cancerous subpopulations (M cells in our model) to enhance the invasive and metastatic behavior of the latter; and (2) the ratio of cancerous to non-cancerous stromal SPARC expression levels, rather than overall tumoral or stromal-only expression, may be a significant determinant of metastatic behavior, as suggested by our immunohistochemical analysis and transcript quantification of metastatic and non-metastatic primary prostate cancer samples. A pro-metastatic function of SPARC endogenously produced by neoplastic cells may be attributed to its increased levels and a paracrine function in metastasis-promoting neoplastic cells and possibly also to differences in one or more properties of SPARC produced by non-neoplastic stromal cells, as suggested by its prominent nuclear localization in a significant proportion of metastatic primary tumors. There is evidence for differential postranslational processing of SPARC in different cell types, potentially leading to differential functions [40], and it remains to be explored if this applies to non-neoplastic *vs*. neoplastic or non-metastatic *vs*. metastatic cancer cells.

In addition to our own previous observations [11], other studies have provided examples of cooperation between heterogeneous tumor cell subpopulations that leads to enhanced metastatic behaviors of tumors [8, 33, 63, 64]. One of these studies has identified non-cancerous fibronectin as a key extracellular protein to support the enhanced invasiveness prompted by cooperating neoplastic cell subpopulations [63]. Our current study is the first to identify a specific extracellular matrix remodeling factor produced by a non-CSC neoplastic cell subpopulation as responsible for instigating the invasion and metastatic behavior of a second subpopulation displaying CSC properties. Matricellular proteins, including osteopontin, tenascin or SPARC, are gaining increasing attention for their roles in shaping local or metastatic niches to either support or prevent the growth and colonization potentials of tumor cells [38, 65, 66]. The observations described here indicate that SPARC can also participate in paracrine interactions between tumor cell subpopulations and influence their metastatic potential.

## Conclusions

Interactions among tumor cell subpopulations with distinct phenotypes and gene programs impact the overall behavior of a given tumor. Our analysis of interactions between clonal subpopulations displaying CSC or non-CSC properties has led to the identification of novel paracrine interactions among such subpopulations through the secretion by a non-CSC subpopulation of the matricellular protein SPARC, which boosts the invasive and metastatic potentials of a CSC-enriched subpopulation. Our findings expand the previously known functions of SPARC in tumor-stromal interactions to include interactions among neoplastic subpopulations that impinge upon the metastatic behavior of tumor cells. Finally, the importance of the production of SPARC by tumor cells, in contrast to that produced by stromal cells, in favoring the metastatic spread of prostate cancer cells is underscored by our finding that SPARC is expressed by primary prostate adenocarcinoma cells from metastatic cases, but not from non-metastatic cases. Based on our observations, we conclude that neoplastic-produced SPARC constitutes a potential tumor progression biomarker and a therapeutic target in advanced prostate cancer.

## Materials and methods

### Cell lines

Luciferase-bearing PC-3M and PC-3S cells were clonally derived from the human cell line PC-3 [11]. Du-145, CWR22v1 and LNCaP cells were obtained from the American Type Culture Collection (Manassas, VA). Cells were grown at 37°C in a 5% CO_2_ atmosphere in RPMI 1640 medium supplemented with 10% fetal bovine serum, L-glutamine, non-essential aminoacids, sodium pyruvate, penicillin/streptomycin (PAA Laboratories, Coelbe, Germany) and geneticin (Santa Cruz Biotechnologies, Santa Cruz, CA).

### Spheroid formation assay

Cells (10^3^/well) were seeded on 24-well Ultra Low Attachment culture plates (Corning Costar, Cambridge, MA) in complete culture medium containing 0.5% methyl cellulose (Sigma-Aldrich, St. Louis, MO), allowed to grow for 14 days and spheroids scored by image acquisition and spheroid area quantification with ImageJ.

### *In vitro*invasiveness assays

Transwell-Matrigel invasion assays were performed as described [11]. To harvest S conditioned medium (S-CM) or M conditioned medium (M-CM), cells were cultured to 70% confluence, at which time the culture medium was replaced with fresh CD-CHO medium (Invitrogen) supplemented with 8 mM glutamine (PAA). After 48 h, conditioned media were collected, centrifuged and filtered through a 0.2 μm filter (VWR Company, Darmstadt, Germany). M cells were analyzed for invasiveness by seeding with S-CM or M-CM (control) on Matrigel-hyaluronic acid-coated Transwell chambers. For co-culture experiments, M cells were loaded with Oregon Green 488 carboxy-DFFDA-SE (Invitrogen) and S cells with Far Red DDAO-SE (Invitrogen), by adding 25 μM of fluorophore to the cell suspensions for 30 min, washed with PBS, and reseeded. Fluorophore-preloaded cells were co-cultured at a 1:1 ratio on Matrigel-Transwell units and scored for invasiveness after 24 h.

### SILAC labeling and sample preparation

M and S cells were cultured in L-lysine and L-arginine-depleted RPMI (Thermo Scientific, Hudson, NH) supplemented with 10% dialyzed FBS, antibiotics (PAA), and either 0.1 mg/mL ^12^C_6_- (M) or 0.1 mg/mL ^13^C_6_- (S), L-lysine and L-arginine (Thermo). The medium was replaced every 2 days, and cells routinely passaged at 80-90% confluence. After 14 days, cells were cultured for 2 days in light (M) or heavy (S) labeled medium without FBS, conditioned mediums collected, centrifuged, filtered through a 0.2 μm filter (VWR) and concentrated using Amicon centrifugal filter devices with a 3-kDa molecular weight cut-off (Millipore, Billerica, MA). Protein content was quantified (Bradford RcDc protein assay; BioRad, Hercules, CA) and 15 μg of each sample were mixed, diluted in 50 mM ammonium bicarbonate and concentrated using an Amicon centrifugal filter device with a 10-kDa molecular weight cut-off (Millipore). The <10 kDa fraction was evaporated to dryness, resuspended in 8 M urea, 50 mM ammonium bicarbonate, reduced with 50 mM dithiothreitol, alkylated with 125 mM iodoacetamide, digested with trypsin and analysed by LC-MS. The >10 kDa fraction was resuspended in loading buffer and subjected to electrophoresis on a 12.5% SDS-polyacrylamide gel.

### Mass spectrometry analysis

After SDS-PAGE and Coomassie blue staining, lanes were split into 10 slices, digested with modified porcine trypsin (Promega, Madison, WI), dried, extracted with formic acid solution and analyzed on an Esquire Ultra IT mass spectrometer (Bruker, Bremen, Germany) coupled to a nano-HPLC system (Ultimate; LC Packings, Amsterdam, The Netherlands). Peptide mixtures were concentrated on a 300 mm i.d., 1 mm PepMap nanotrapping column and loaded onto a 75 mm i.d., 15 cm PepMap nanoseparation column (LC Packings). Peptides were eluted by an acetonitrile gradient (0–60% B in 150 min, where B is 80% acetonitrile, 0.1% formic acid in water; flow rate ca. 300 nL/min) through a PicoTip emitter nanospray needle (New-Objective, Woburn, MA) onto the nanospray ionization source of the IT mass spectrometer. MS/MS fragmentation (1.9 s, 100–2800 m/z) was performed on three of the most intense ions, as determined from a 1.2 s MS survey scan (310–1500 m/z), using a dynamic exclusion time of 1.2 min for precursor selection and excluding single-charged ions.

### Protein identification and data analysis

Protein identification and quantification was performed using Protein Scape 2.1 and WARP-LC 1.2 (Bruker). Proteins were identified using Mascot (Matrix Science, London, UK) on the SwissProt human protein database. MS/MS spectra were searched with a 1.5 Da precursor mass tolerance, 0.5 Da fragment tolerance, 1 missed cleavage maximum trypsin specificity, cysteine carbamidomethylation set as fixed modification and methionine oxidation and the N-terminal and Lys and Arg SILAC labels as variable modifications. Positive identification criterion was set as an individual Mascot score for each peptide MS/MS spectrum above the homology threshold score. False positive rates for Mascot protein identification were measured by searching a randomized decoy database [67], and estimated to be under 4%. For relative protein quantification, H/L ratios were calculated averaging the measured H/L ratio for the observed peptides, after discarding outliers. For selected proteins of interest, quantitative data obtained from the automated Protein Scape analysis were manually curated. For further protein analysis, UniProtKB (http://www.uniprot.org) and GeneCards databases (http://www.genecards.org) were used.

### Western blotting

Samples were resuspended in Laemmli buffer (100 mM dithiothreitol, 50 mM TrisCl pH 6.8, 2% SDS, 0.1% bromophenol blue, 10% glycerol) and boiled for 5 min. Equal amounts of protein were resolved by SDS-PAGE. After electrophoresis, proteins were transferred onto fluorescent-PVDF membranes (Immobilon-FL, Millipore) for 2–4 h. Blots were washed, blocked with blocking buffer (Odyssey, LI-COR), incubated overnight with primary antibody diluted in blocking buffer-PBS (or TBS)-Tween (0.1%) (1:1), washed in PBS-T (or TBS-T) and incubated for 1 h with fluorescent secondary antibody IRDye (LI-COR Biosciences, Lincoln, NE). After final washes in PBS-T (or TBS-T), the membranes were scanned using the Odyssey Infrared Imaging System (LI-COR Biosciences, Lincoln, NE). Antibodies to the following antigens were used: SPARC (1:200) (H-90, Santa Cruz), PAI-1 (1:200) (C-9, Santa Cruz), AKT1 (1:100) (C-20, Santa Cruz), phospho-AKT1 (1:100) (pSer473, Santa Cruz), ERK1/2 (1:200) (H-72, Santa Cruz), phospho-ERK1/2 (1:100) (pThr177/pThr160, Santa Cruz), ILK (1:100) (E-2, Santa Cruz), phospho-ILK (1:100) (pThr173, Santa Cruz) and β-tubulin (1:2,000) (Sigma).

### Lentiviral shRNA production and transduction

pLKO.1-Puro plasmids for control (shC002) and SPARC-targeting shRNAs TRCN0000008709 (sh8709) and TRCN0000008711 (sh8711) were from Sigma-Aldrich. Doxycycline-inducible SPARC-targeting V2THS_153399 (sh3399) was from Thermo. Each of these plasmids was co-transfected in HEK293T cells with pVSVG and pCMVΔR8.91 (Clontech, Mountain View, CA) using X-tremeGENE9 (Roche). Supernatants were collected for the following 48 h and filtered through 0.45 μm methylcellulose filters (Millipore). Viral particles were concentrated by ultracentrifugation on 20% sucrose gradients. Target cells were transduced in the presence of polybrene (8 μg/mL; Sigma-Aldrich), and selected with 1 μg/mL puromycin (Sigma-Aldrich) for 7 days.

### Production and purification of recombinant human SPARC

HEK 293T cells bearing integrated copies of a human SPARC expression vector [68] were grown in DMEM supplemented with 10% FBS and 3 μg/mL puromycin. Confluent cells were grown in serum- and puromycin-free medium and conditioned medium harvested every 2 or 3 days for 7–8 harvests and concentrated 10-fold on 30,000 Da cut-off Amicon centrifugal filter devices (Millipore) with simultaneous exchange into starting buffer (20 mM MOPS, 200 mM LiCl, pH 6.5). Concentrates were bound to a Maxi Anion (Q) Spin Column (Thermo) equilibrated with starting buffer and eluted with 30% elution buffer (20 mM MOPS, 400 mM LiCl, pH 6.5). SPARC-containing fractions, monitored by SDS-PAGE, were concentrated and dialyzed against Hank’s balanced salt solution (HBSS).

### Immunodepletion experiments

S-CM was obtained as described above and subjected to immunoprecipitation using 2 μg/mL rabbit anti-SPARC (H-90, Santa Cruz) or anti-PAI-1 (C-9, Santa Cruz) plus pre-washed protein G-Sepharose (GE Healthcare, Buckinghamsire, UK) at 4°C for 2 h. Subsequently, samples were centrifuged and supernatants collected and used in invasiveness assays. Pelleted beads were washed three times with PBS and resuspended in Laemmli buffer. Aliquots from supernatant and resuspended pelleted beads were processed for Western blotting analysis as above.

### Orthotopic prostate model and bioluminescence imaging (BLI)

M and S.sh3399 cells were transduced with the pRRL-Luc-IRES-EGFP lentiviral vector [11] for the constitutive expression of the firefly luciferase gene to generate M.Fluc and S.Fluc.sh3399 cells, respectively. M.Fluc cells (1 × 10^5^), or a mix of M.Fluc (1 × 10^5^) with S.Fluc.sh3399 (1 × 10^5^) cells, pretreated or not *in vitro* with doxycycline (1 μg/mL; Sigma-Aldrich), resuspended in 30 μL sterile PBS, were inoculated into the dorsal prostates of 6-week-old SCID-Beige mice. Doxycycline (1 mg/mL) was administered *ad libitum* in drinking water containing 25 mg/mL sucrose (Sigma-Aldrich) to induce the expression of shSPARC. BLI was performed with the IVIS Spectrum Imaging System (Perkin Elmer Life Science, Boston, MA), and images and measurements were acquired and analyzed using the Living Image 4.3.1 software (Perkin Elmer). For *in vivo* BLI, animals were anesthetized with 1-3% isoflurane (Abbott Laboratories, IL) and injected i.p. with 150 mg/kg of D-luciferin (Promega) in sterile PBS. For *ex vivo* BLI, mice were injected i.p. with 150 mg/kg D-luciferin prior to euthanasia. Immediately postmortem, organs of interest were placed individually into separate wells with 300 μg/mL D-luciferin, imaged and quantified as above. These experiments were performed at the CIBER-BBN *In Vivo* Experimental Platform.

### RNA isolation, reverse transcription and real-time RT-PCR

RNA purification and reverse transcription were performed as described [11]. Real-time quantitative PCR assays were performed on a LightCycler 480 instrument (Roche) and analyzed with the LightCycler 480 Software release 1.5.0. The Universal Probe Library system (UPL) (Roche) was used to quantify transcripts. Probes and sequences are shown in Additional file 2: Table S2. RN18S1 amplification levels were used as an internal reference, and relative transcript quantification determined by the ∆∆Cp method. Tissue samples were procured through the Hospital Clínic-IDIBAPS Biobank, a Generalitat de Catalunya authorized biobank registered at the Instituto de Salud Carlos III, and thus sample collection and processing fulfilled all ethical and legal requirements.

### Tissue microdissection and transcript quantification

Eight formalin-fixed paraffine-embedded samples were used for laser microdissection, 4 from non-metastatic cases and 4 from metastatic cases. Eight-μm sections from each sample were mounted onto plastic membrane slides (Leica Microsystems, Germany), stained with hematoxylin-eosin, air dried and stored at -80°C until use. Laser microdissection was performed with the Leica LMD7000 System (Leica). Approximately 3 mm^2^ of either tumoral epithelium or stroma were collected separately for each sample. RNA isolation was performed with the RNeasy FFPE Kit (Qiagen) and reverse transcribed with the High-Capacity cDNA Reverse Transcription Kit (Life Technologies). cDNA was preamplified (14 cycles) with the TaqMan PreAmp Master Mix (Life Technologies). SPARC transcript levels were quantified using a TaqMan assay (Hs00234160_m1) and normalized to β-2 microglobulin (B2M) transcript levels (assay Hs00984230_m1). Real-time PCR assays were performed and analyzed as described above.

### Immunohistochemistry

A total of 30 samples were used for immunohistochemical detection of SPARC protein, 14 from non-metastatic and 16 from metastatic cases. Two μm thick sections were mounted on xylaned glass slides (DAKO, Glostrup, Denmark) and processed for antigen retrieval in citrate buffer pH 6 for 20 minutes, incubated for 1 h with rabbit-anti-SPARC (1:300) (H-90, Santa Cruz) and reactions revealed with the Bond Polymer Refine Detection System (Leica, Wetzlar, Germany). The staining was scored as the percentage of positive cells with null, weak, moderate or strong intensities, scoring separately the epithelial and stromal compartments of each sample. Images were captured with an Olympus BX-51 microscope equipped with an Olympus DP70 camera.

### Immunocytochemistry

Cells were seeded on sterile round glass coverslips, allowed to attach for 24 to 48 h, washed with PBS, fixed with cold 4% paraformaldehyde for 20 min, permeabilized with 1% Triton X-100 in PBS, blocked for 30 min with blocking buffer (3% BSA, 1% Triton X-100 in PBS), incubated with primary antibody (anti-SPARC, 1:50 in blocking buffer) for 2 h at room temperature, washed 3× with blocking buffer, and incubated for 30 min with Alexa Fluor 488-conjugated rabbit-anti-mouse antibodies (Life Technologies; 1:1,000 in blocking buffer), Alexa Fluor 555-conjugated phalloidin (Life Technologies; 1:10,000) and 2-(4-amidinophenyl)-6-indolecarbamidine dihydrochloride, 4′,6-Diamidino-2-phenylindole (DAPI) dihydrochloride (Sigma; 0.1 μg/mL). After washes, samples were mounted on glass slides with Mowiol 18–88 (Sigma) and visualized under a Leica SP5 confocal microscope (Leica, Wetzlar, Germany).

### Plasma SPARC quantification by ELISA (Enzyme-Linked ImmunoSorbent Assay)

Plasma samples were collected from 4 healthy controls (blood PSA <4 ng/mL) and 10 non-metastatic and 14 metastatic prostate cancer patients, using EDTA as anticoagulant. Blood from patients was extracted prior to prostatectomy. No patients or controls were under systemic treatment at the time of blood extraction. Quantification of SPARC protein levels was performed using Quantikine ELISA Human SPARC Immunoassay (R&D Systems, Abingdon, UK). Tissue and blood samples were procured through the Hospital Clínic-IDIBAPS Biobank, a Generalitat de Catalunya authorized biobank registered at the Instituto de Salud Carlos III, and thus sample collection and processing fulfilled all ethical and legal requirements.

### Statistical analysis

A Student’s *t*-test was applied to two-way comparisons of data sets from *in vitro* experiments. A non-parametric Mann–Whitney test was applied to SPARC level determinations in tissues and blood and for bioluminescent data from xenograft experiments. The significance threshold was established at *P* < 0.05, and significance levels were schematically assigned * (0.01 ≤ *P* < 0.05), ** (0.001 ≤ *P* <0.01) or *** (*P* < 0.001).

## Electronic supplementary material

Additional file 1: **Contains: Tables S1, S3 and S4 and Figures S1-S13 with legends.** (PDF 894 KB)

Additional file 2: Table S2: List of proteins identified in the mixture of S-conditioned medium (“heavy” labeled proteins) and M-conditioned medium (“light” labeled proteins). (XLS 131 KB)

## Abbreviations

BLI: Bioluminescence imaging
CM: Conditioned medium
CSC: Cancer stem cell
ELISA: Enzyme-linked immunosorbent assay
IKK-β: Inhibitor of nuclear factor kappa-B kinase subunit beta
i.p.: Intraperitoneal
M: PC-3M
MAPK: Mitogen-activated protein kinase
NF-κB: Nuclear factor kappa-light-chain-enhancer of activated B cells
PAI-1: Plasminogen activator inhibitor-1
PCR: Polymerase chain reaction
PDGF: Platelet-derived growth factor
PTEN: Phosphatase and tensin homolog
qPCR: Quantitative polymerase chain reaction
rhSPARC: Recombinant human SPARC
S: PC-3S
SDS-PAGE: Sodium dodecyl sulfate polyacrylamide gel electrophoresis
SILAC: Stable isotope labeling by aminoacids in cell culture
SPARC: Secreted protein, acidic, and rich in cysteine
TGF-β: Transforming growth factor beta.
