# Supplementary material for: SPARC mediates metastatic cooperation between CSC and non-CSC prostate cancer cell subpopulations
Source: Mol Cancer. 2014 Oct 21;13:237. doi: 10.1186/1476-4598-13-237 (PMC4210604; doi:10.1186/1476-4598-13-237)
Supplement: Supplementary file 1 — Additional file 1: Contains: Tables S1, S3 and S4 and Figures S1-S13 with legends. (PDF 894 KB) [file 12943_2014_1437_MOESM1_ESM.pdf]

SPARC mediates metastatic cooperation between CSC and non-CSC prostate cancer cell subpopulations

Francesca Mateo, Óscar Meca-Cortés, Toni Celià-Terrassa, Yolanda Fernández, Ibane Abasolo, Lourdes Sánchez-Cid, Amaia Sagasta, Leonardo Rodríguez-Carunchio, Raquel Bermudo, Mònica Pons, Verónica Cánovas, Mercedes Marín-Aguilera, Lourdes Mengual, Antonio Alcaraz, Simó Schwartz Jr., Begoña Mellado, Kristina Y. Aguilera, Rolf Brekken, Pedro L. Fernández, Rosanna Paciucci and Timothy M. Thomson

Contents:

Supplementary Tables1, 3 and 4

Supplementary Figures 1-13 with legends

Supplementary Table 1. Pathway inhibitors and concentrations used in Transwell-Matrigel assays.

INHIBITOR	TARGET	Working concentration	Approx % of inhibition of invasiveness
GF109203	PKC	1 μM	0%
SB431542	TGF-β	10 μM	0%
BAY 11-7085	IκBα	1 μM	10%
DAPT	NOTCH	10 μM	20%
AG1478	EGFR	2.5 μM	20%
Cyclopamine	Hedgehog	5 μM	20%
Rapamycin	mTOR	1 μM	20%
SB203580	p38	10 μM	40%
LY294002	PI3K	10 μM	50%
PD98059	MEK1	10 μM	60%
SC-514	IKKβ	10 μM	60%
Wortmannin	PI3K	250 nM	60%
PP1	Src	10 μM	60%

Supplementary Table 3. Primers and UPL probes used for real-time RT-PCR

GENE	UPL PROBE		OLIGONUCLEOTIDES 5'->3'
RN18S1	#40	FW<br>REV	ggagagggagcctgagaaac<br>tcgggagtgggtaatttgc
SPARC	#77	FW<br>REV	gtgcagaggaaaccgaagag<br>tgtttgcagtggtggttctg
PAI-1	#19	FW<br>REV	aaggcacctctgagaacttca<br>cccaggactaggcaggtg
SODE	#19	FW<br>REV	cttggaggagctggaaaggt<br>gagcaggcaggaacacagta
CALR	#9	FW<br>REV	ctatgataactttggcgtgctg<br>ctcctcagcgtatgcctcat
PTX3	#58	FW<br>REV	tgtatgtgaatttggacaacgaa<br>cattccgagtgctcctgac

Supplementary Table 4. Immunohistochemical staining for SPARC in primary prostate cancer samples.

ID[a]	LN Status	Gleason[b]	Stromal	Epithelial[c]
24	0	3 (6)	100	0
25	0	3, 4 (7)	100	0
26	0	3 (6)	100	40 (C)
27*	0	3 (6)	100	0
28*	0	3, 4 (7)	95	0
32	0	3 (6)	100	0
33*	0	3, 4 (7)	100	0
35	0	3 (6)	80	0
39	0	3 (6)	100	0
53	0	3, 4 (7)	90	0
54	0	3, 4 (7)	100	0
56	0	3 (6)	100	0
57	0	3, 4 (7)	100	0
58*	0	5 (10)	100	0
29*	1	4, 5 (9)	90	50 (N)
30*	1	4, 5 (9)	95	70 (N)
31*	1	5 (10)	100	65 (C+N)
34	1	4, 5 (9)	100	80 (C+N)
36	1	5 (10)	100	80 (C+N)
37	1	3 (6)	100	10 (C+N)
38*	1	4, 5 (9)	95	80 (C+N)
40	1	3 (6)	100	0
41	1	3, 4 (7)	80	0
59	1	5 (10)	90	80 (N)
60	1	4, 5 (9)	100	90
61	1	4, 5 (9)	100	95 (C)
62	1	3, 4 (7)	95	95 (C)
64	1	4, 5 (9)	100	90 (C+N)
65	1	5 (10)	1	80 (C+N)
66	1	4, 5 (9)	95	100 (C)

a Asterisks indicate samples analyzed for SPARC mRNA transcript levels in microdissected stromal/epithelial components (Figure 6).

b Gleason pattern indexes assigned to two representative areas in each sample. A single digit indicates same index for the two areas. Parenthesis: combined Gleason scores.

c Percentage of cells with unequivocal positive staining for SPARC (intensity range 1-3; not shown) in epithelial components. Parenthesis: predominantly cytoplasmic (C), nuclear (N) or cytoplasmic and nuclear (C+N) staining.

Supplementary Figure 1

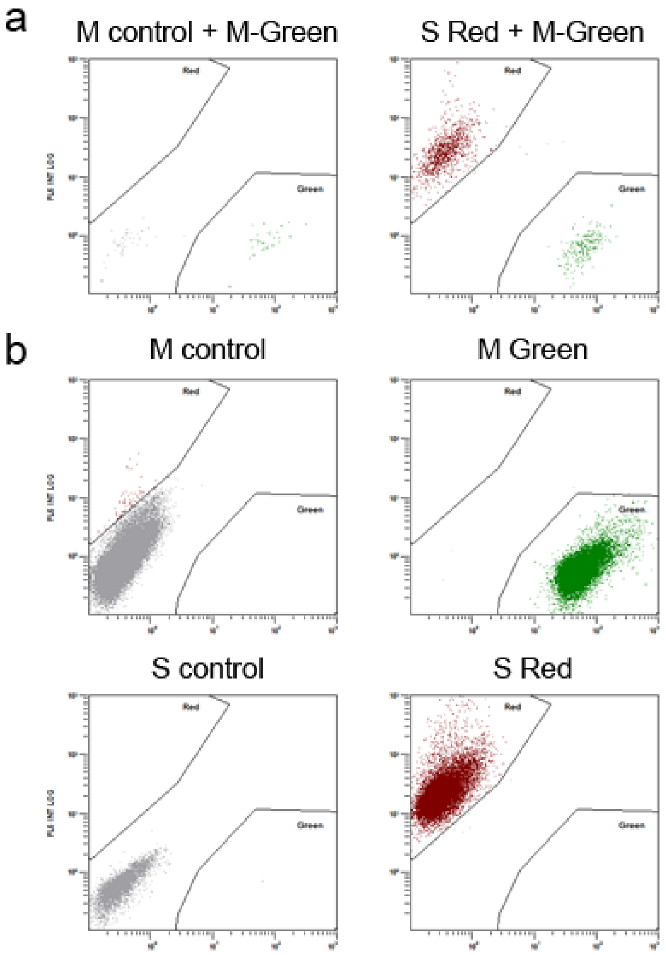

<u>**Supplementary Figure 1**</u>**. Flow cytometry quantification of the invading cells from the Transwell-Matrigel experiment shown in Figure 1c**. M cells were independently labeled with Oregon Green 488 carboxy-DFFDA-SE and S cells with Far Red DDAO-SE. After labeling, M-Green cells were co-cultured with the same number of unlabeled M cells or S-Red cells on Transwell-Matrigel inserts. After 24 h, invading cells were collected at the bottom chamber and analyzed by flow cytometry. (**a**) Left: Dot-plot showing the invading populations of unlabeled M cells and M-Green cells. Right: Dot-plot depicting the populations of invading S-Red and M-Green cells. When M-Green cells are co-cultured with S-Red cells they become more invasive than in the control situation (M-Green cells co-cultured with unlabeled M cells). (**b**) Control dot-plots used for gating M-Green and S-Red cells.

Supplementary Figure 2

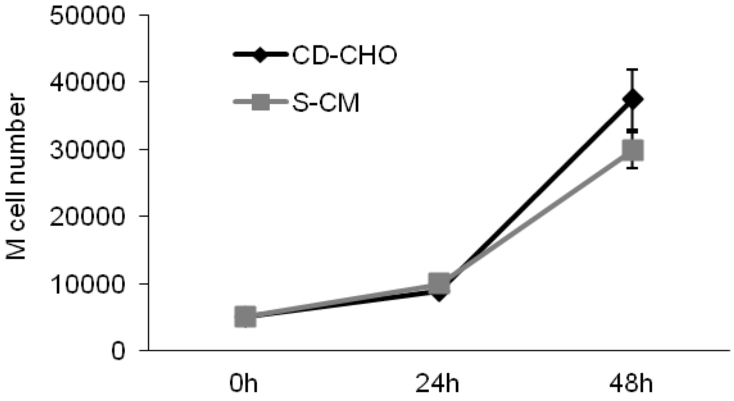

Supplementary Figure 2. S-CM does not augment the proliferation of M cells. M cells (5,000/well) were plated in 96-well plates in the presence of control (CD-CHO) or S conditioned medium (S-CM) and cell growth determined by the MTT colorimetric assay. Experiments were performed in sextuplicates and the data shown are cell number at each time-point ± SD. Cell numbers were extrapolated from a standard curve performed in parallel.

Supplementary Figure 3

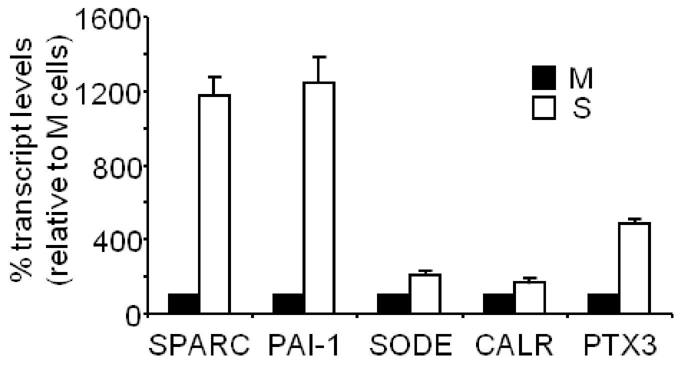

<u>Supplementary Figure 3</u>**. SPARC and PAI-1 transcripts are expressed in S cells at much higher levels than in PC-3Mc cells**. Transcript levels of the indicated genes were quantified by real-time RT-PCR with the UPL system, on a LightCycler 480 instrument. RPL18 (S18) levels were used as internal references and the ΔΔCt method was applied to normalize against values determined for M cells. Values are plotted as percent transcript levels in S cells relative to M cells. Experiments were performed in triplicate and shown are mean values ± SD.

Supplementary Figure 4

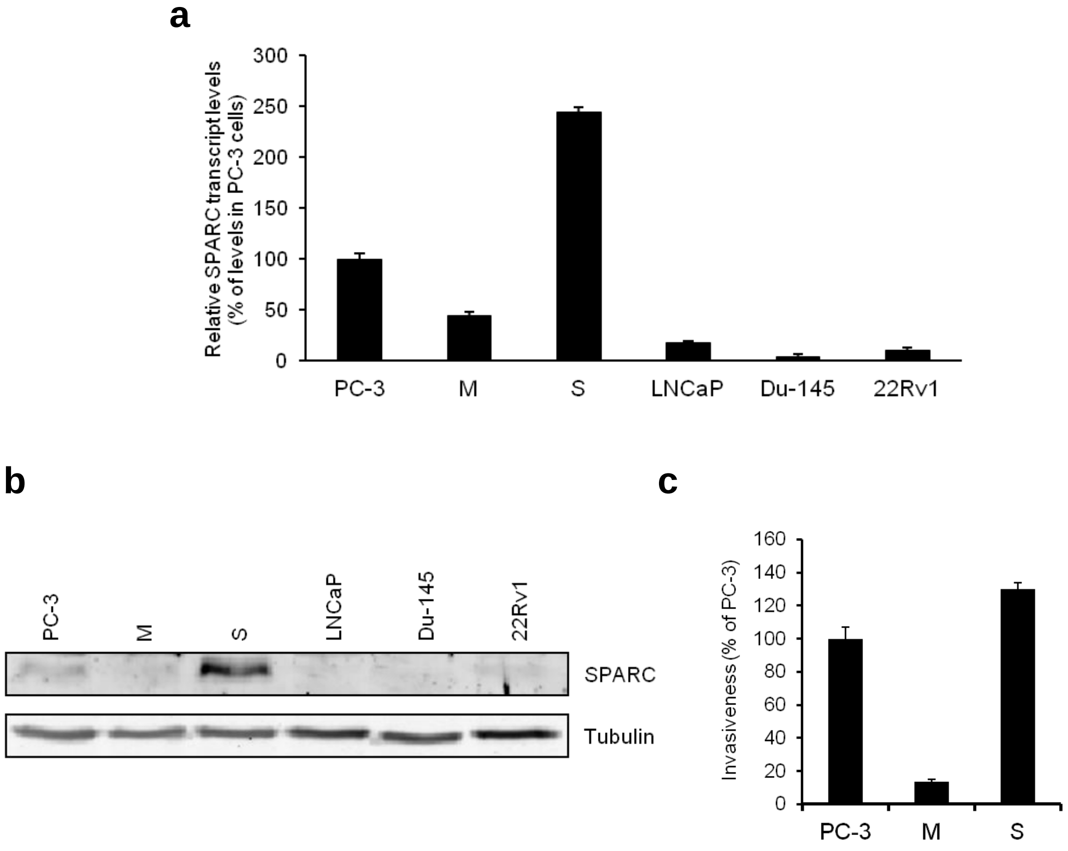

<u>Supplementary Figure 4</u>. (**a**) SPARC transcript levels in different cell lines were quantified by real-time RT-PCR. RPL18 (S18) levels were used as internal references and the ΔΔCt method was applied to normalize against values determined for PC-3 parental cells. Values are plotted as percent transcript levels in different cell lines relative to PC-3 parental cell line. Experiments were performed in triplicates and shown are mean values ± SD. (**b**) Western blotting experiments performed on cell lysates indicate that SPARC protein levels are highest in S cells compared to the rest of cell lines analyzed, which correlates with SPARC transcript levels depicted in (**a**). (**c**) Transwell-Matrigel assay comparing the invasiveness of parental PC-3 cells and the clonal lines PC-3Mc (M) and PC-3S (S).

Supplementary Figure 5

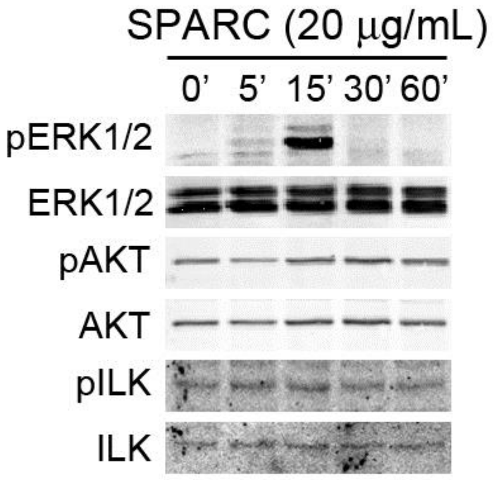

<u>**Supplementary Figure 5**</u>**. Induction of the MAPK pathway in M cells by SPARC.** Overnight serum-starved M cells were incubated with basal medium without (0’) or with SPARC (20 µg/mL) for the indicated times and submitted to Western blotting analysis with antibodies to total or phospho-ERK1/2 (pThr177/Thr160), AKT (pSer473) or ILK (pThr173). The images for pILK and ILK are highly enhanced for visualization because of their weak signals.

Supplementary Figure 6

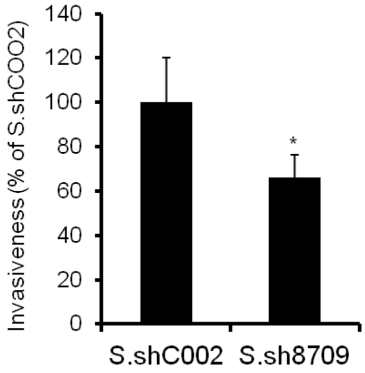

Supplementary Figure 6. Transwell-Matrigel assays comparing the invasive ability of parental S.shC002 (control) cells and S.sh8709 (SPARC-KD) indicate that depletion of SPARC in S cells impairs their invasiveness. Experiments were performed in triplicates and data shown are percentages of invasiveness relative to control cells ± SD. Asterisk denotes statistically significant differences (two-tailed Student's t-test).

Supplementary Figure 7

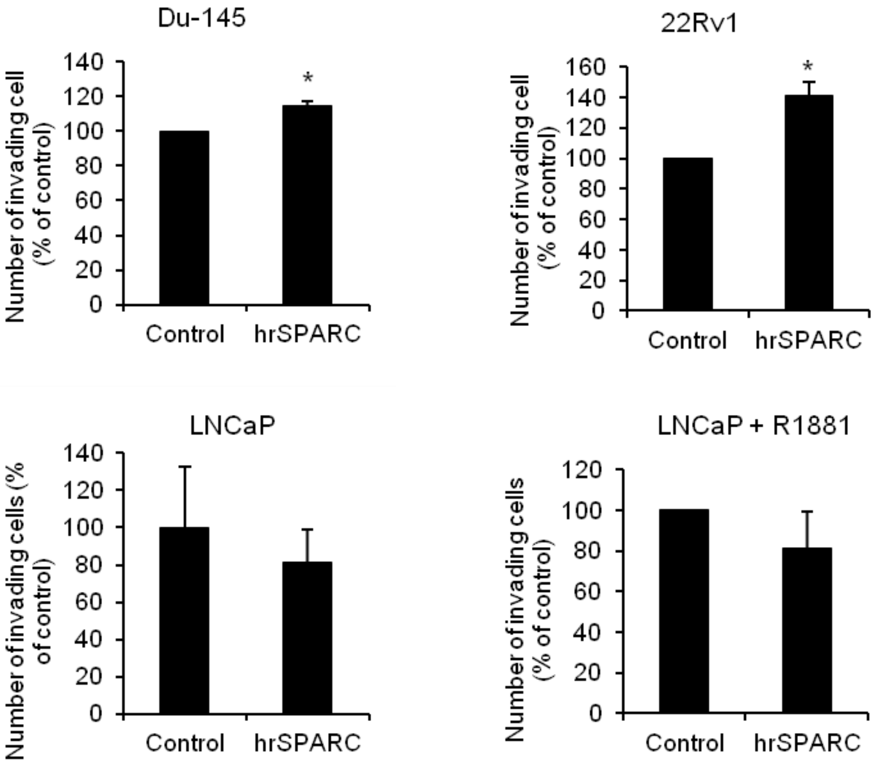

Supplementary Figure 7. Exogenous SPARC enhances the invasiveness of Du-145 and 22Rv1 cells but not LNCaP cells. Transwell-Matrigel assay comparing the relative invasiveness of Du-145, 22Rv1 and LNCaP, cell lines under standard conditions or treated with 20 µg/mL of recombinant human SPARC protein. In the case of the androgen-dependent cell line LNCaP, experiments were performed in the absence (standard conditions) or presence of the androgen agonist R1881 (1 nM), giving similar results. All experiments were performed in quadruplicate and the data shown are percentages of invasiveness relative to control ± SD. Asterisks denote statistically significant differences (two-tailed Student’s t-test).

Supplementary Figure 8

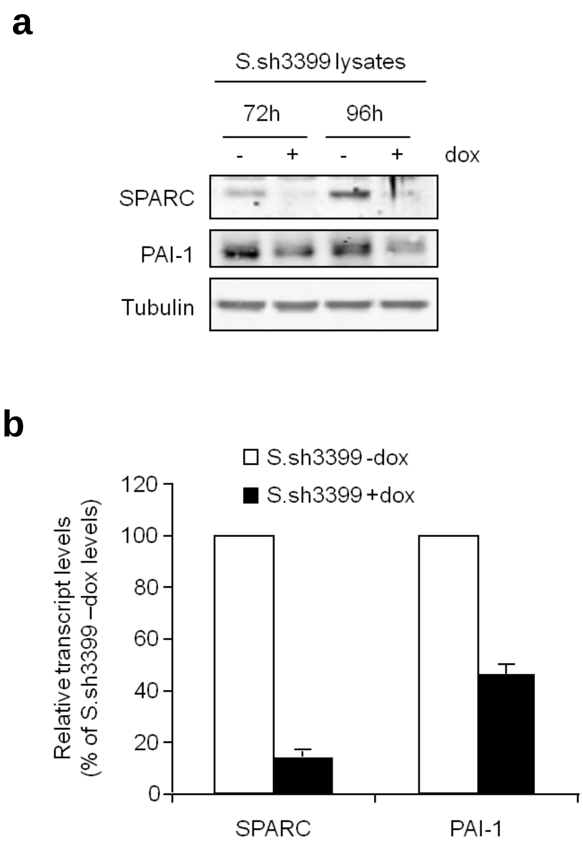

<u>Supplementary Figure 8</u>**. Knock down of SPARC in PC-3S cells is accompanied with a downregulation of PAI-1**. (**a**) Western blot analysis of PAI-1 and SPARC levels following doxycycline-induced knockdown of SPARC in S.sh3399 cells. (**b**) Quantification by real-time RT-PCR of PAI-1 and SPARC transcript levels following doxycycline-induced knockdown of SPARC in S.sh3399 cells.

Supplementary Figure 9

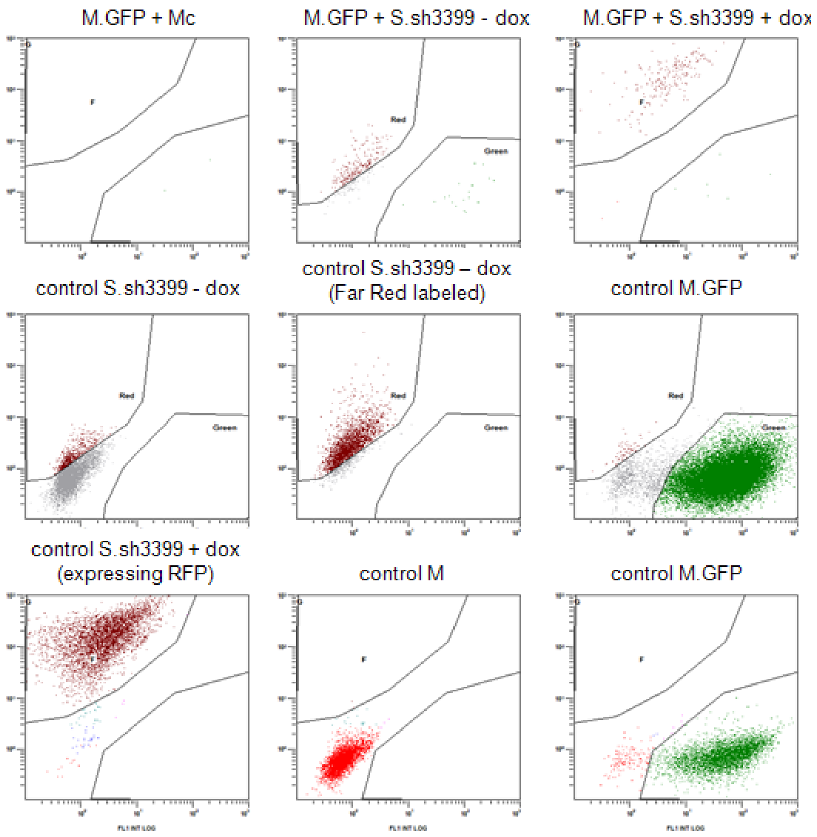

<u>**Supplementary Figure 9**</u>**. Flow cytometry quantification of invading cells from the Transwell-Matrigel experiment shown in Figure 4d**. The following cells were used: unlabeled M, M.GFP, Far Red DDAO-SE-labeled S.sh3399 untreated with doxycycline and S.sh3399 cells treated with 1 µg/mL doxycycline for 24 h prior to the Transwell assay. M.GFP cells were co-cultured with the same number of unlabeled PC-3M cells or S.sh3399 cells (not-treated/treated with doxycycline) for 24 h on Transwell-Matrigel inserts. After 24 h, invading cells were collected from the bottom chamber and analyzed by flow cytometry. (**a**) Left: Dot-plot showing the invading populations of unlabeled M cells and M.GFP cells. Middle: Dot-plot showing the invading populations of M.GFP and S.sh3399 cells (not-treated with doxycycline and Far Red-labeled). Right: Dot-plot depicting the populations of invading M.GFP and S.sh3399 cells (treated with doxycycline, RFP-expressing). When M.GFP cells are co-cultured with S.sh3399 – dox cells, they become more invasive than in the control situation (M.GFP + M). This enhanced invasiveness is largely lost upon co-culture with S.sh3399 + dox (S cells knocked-down for SPARC). (**b**) and (**c**) Control dot-plots used for gating M.GFP and Red-labeled S cells.

Supplementary Figure 10

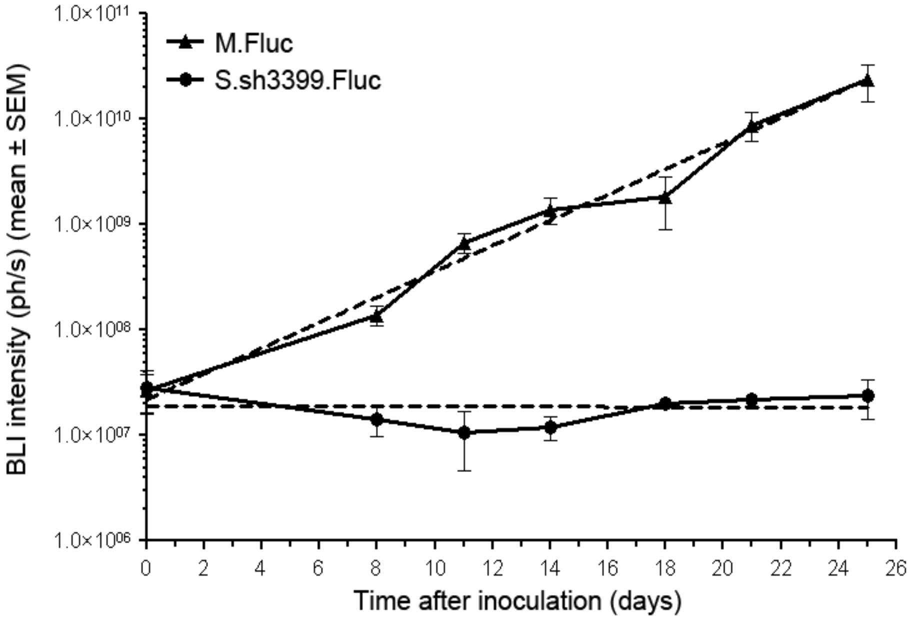

<u>**Supplementary Figure 10**</u>**. PC-3S.sh3399 cells do not grow detectable tumors 25 days after orthotopic implantation**. Dorsal prostates of SCID-Beige mice were inoculated with $1x10^5$ M.Fluc or S.sh3399.Fluc cells and tumor growth monitored by bioluminescent quantification and imaging as described in Materials and Methods. Dotted lines represent non-linear fitting to the exponential growth equation $Y = Y_0^{(kX)}$.

Supplementary Figure 11

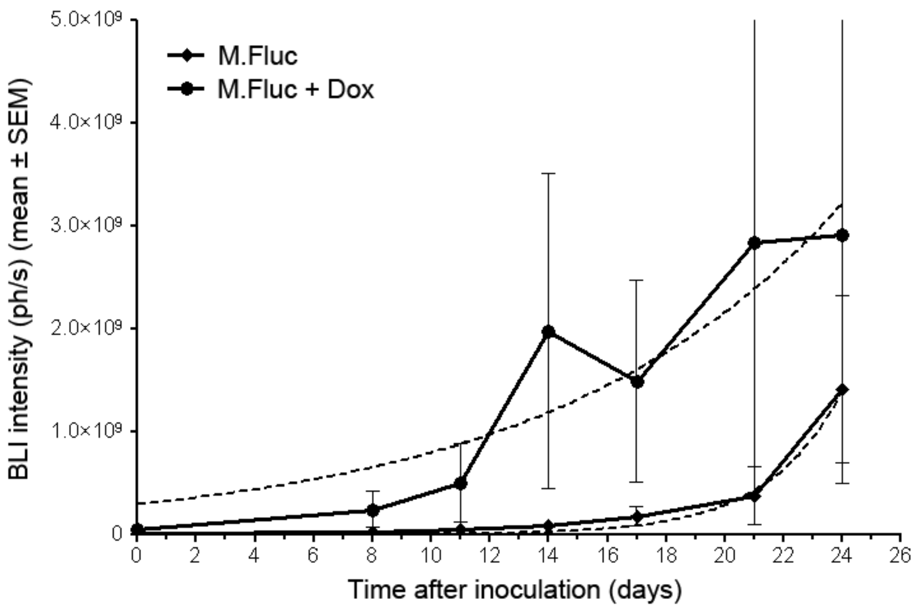

Supplementary Figure 11. Doxycycline does not inhibit PC-3Mc tumor growth. Dorsal prostates of SCID-Beige mice were inoculated with $1x10^5$ M.Fluc cells and one group was given plain water (M.Fluc) and a second group doxycycline (1 mg/mL) in their drinking water (M.Fluc + Dox). Tumor growth was monitored by bioluminescent quantification and imaging as described in Materials and Methods. Dotted lines represent non-linear fitting to the exponential growth equation $Y = Y_0^{(kX)}$. The apparent differences in growth rates observed between the two groups of animals are not statistically significant.

Supplementary Figure 12

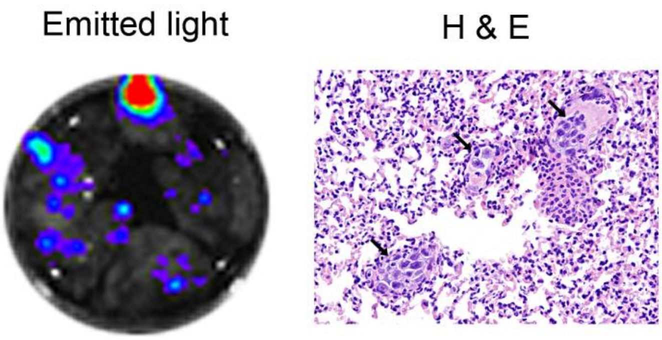

Supplementary Figure 12. ***Ex vivo*** **BLI-positive lungs containing tumor cells observed by H&E staining**. The images correspond to a representative animal implanted in the dorsal prostate with M cells admixed with S.sh3399 cells (without doxycycline administration), sacrificed 24 days after implantation, their lungs removed postmortem and quantified and imaged for emitted light after incubation with D-luciferine, as detailed in Materials and Methods (left panel). The same lungs were formalin-fixed and paraffin-embedded and sections stained for hematoxylin-eosin (right panel). Arrows: nests of tumor cells in pulmonary parenchyma.

Supplementary Figure 13

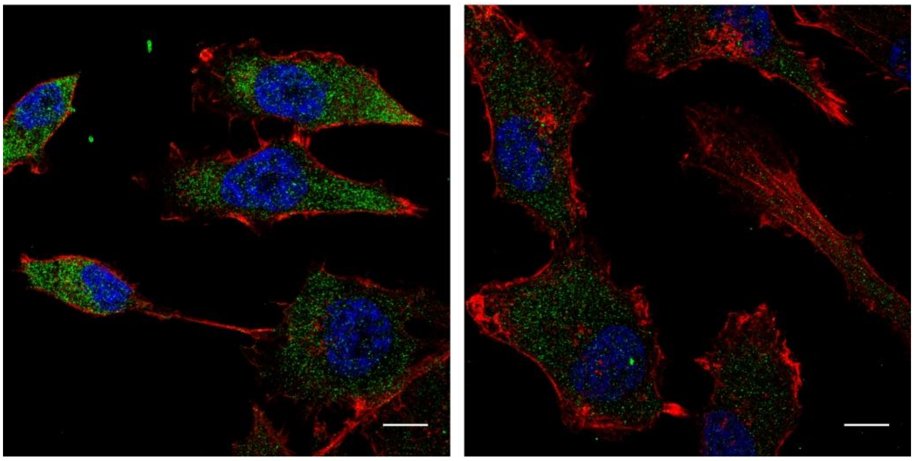

Supplementary Figure 13. Intracellular localization of SPARC in M cells. Overnight serum-starved M cells were incubated for 2 h with conditioned medium from SPARC-producing S cells (S-CM) or from SPARC-knockdown S cells (S/SPARCkd-CM), fixed (4% paraformaldehide), permeabilized (1% Triton X-100) and submitted to indirect immunofluorescence for SPARC (green fluorescence). Cell countour and actin stress fibers (f-actin) were visualized by incubation of samples with with Alexa Fluor 555-phalloidin (red) and nuclei were visualized by staining DNA with DAPI (blue). Fluorescent images were captured with a Leica SP5 confocal microscope at equal laser excitation power and emission acquisition parameters for all samples.
